# Supplementary material for: 2′-O-Methyl at 20-mer Guide Strand 3′ Termini May Negatively Affect Target Silencing Activity of Fully Chemically Modified siRNA
Source: Mol Ther Nucleic Acids. 2020 May 15;21:266–77. doi: 10.1016/j.omtn.2020.05.010 (PMC7327867; doi:10.1016/j.omtn.2020.05.010)
Supplement: Document S1. Figures S1–S7 and Tables S1–S9 [file mmc1.pdf]

OMTN, Volume *21*

Supplemental Information

2′-*O*-Methyl at 20-mer Guide Strand 3′ Termini May Negatively Affect Target Silencing Activity of Fully Chemically Modified siRNA

Sarah M. Davis, Jacquelyn Sousa, Lorenc Vangjeli, Matthew R. Hassler, Dimas Echeverria, Emily Knox, Anton A. Turanov, Julia F. Alterman, and Anastasia Khvorova

Supplemental Figures

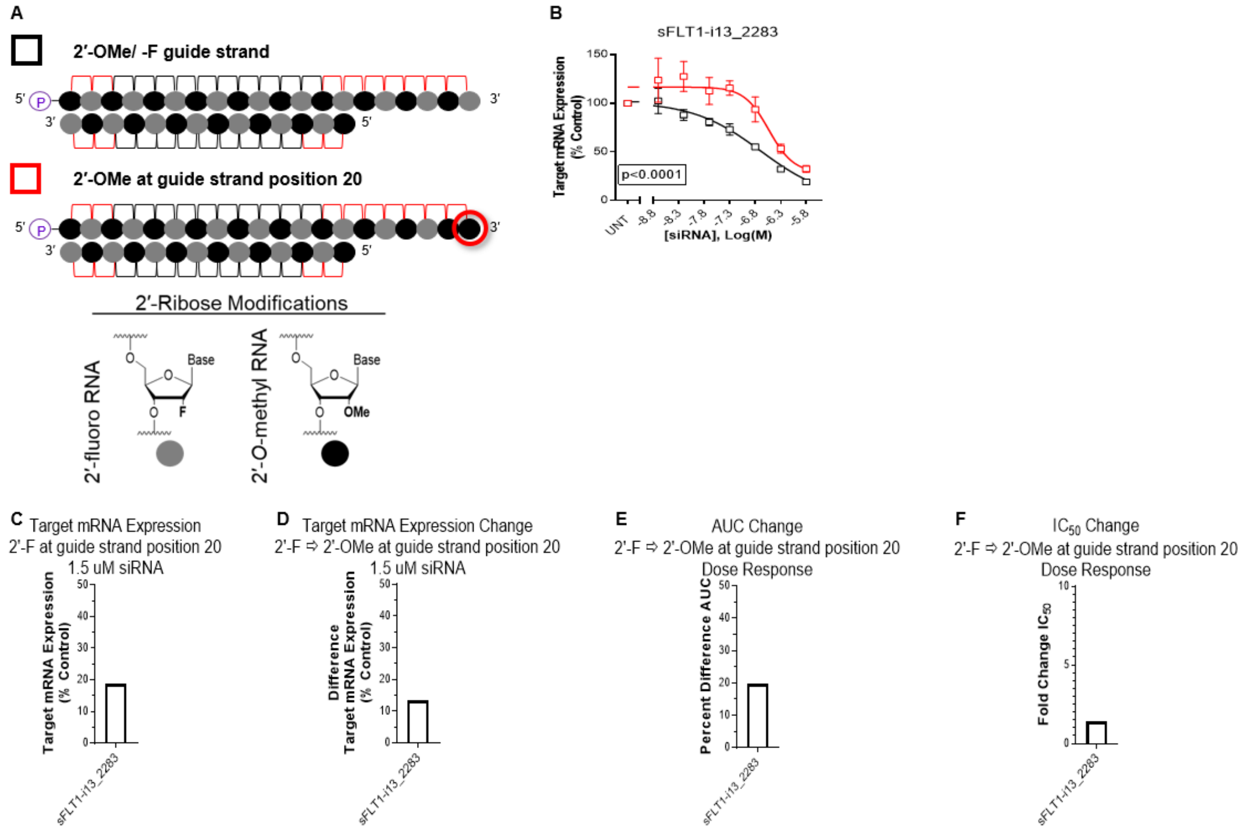

Figure S1. 2′-*O*-methyl at 3′ termini of 20-mer guide strands decreases activity of fully modified, asymmetric siRNAs with 14-mer passenger strands. (A) Schematic representations of chemical modification scaffolds used; symbols next to each schematic are used in graphs shown in (B). Legend shows corresponding chemical structures for 2′-ribose modifications. (B) Dose response results (n=3, mean ± SD). Target name and start site of target sequence indicated in each graph. WM-115 cells were treated with siRNAs at concentrations shown for 72 hours. mRNA levels were measured using the Quantigene 2.0 RNA Assay and calculated as a percent of those from untreated cell controls. p-values displayed on each graph calculated by two-way ANOVA. (C) Target mRNA expression with 1.5 µM of each siRNA with 2′-F at guide strand position 20. (D) Differences in target mRNA expression with 1.5 µM of each siRNA when 2′-OMe replaces 2′-F at guide strand position 20. (E) Percent differences in AUCs. (F) Fold changes in IC_{50} values, calculated by dividing the value obtained from siRNAs with 2′-OMe at guide strand position 20 by the value obtained from siRNAs with 2′-F at guide strand position 20. (D, E, F) Positive values indicate increases in values when 2′-OMe replaces 2′-F at guide strand position 20.

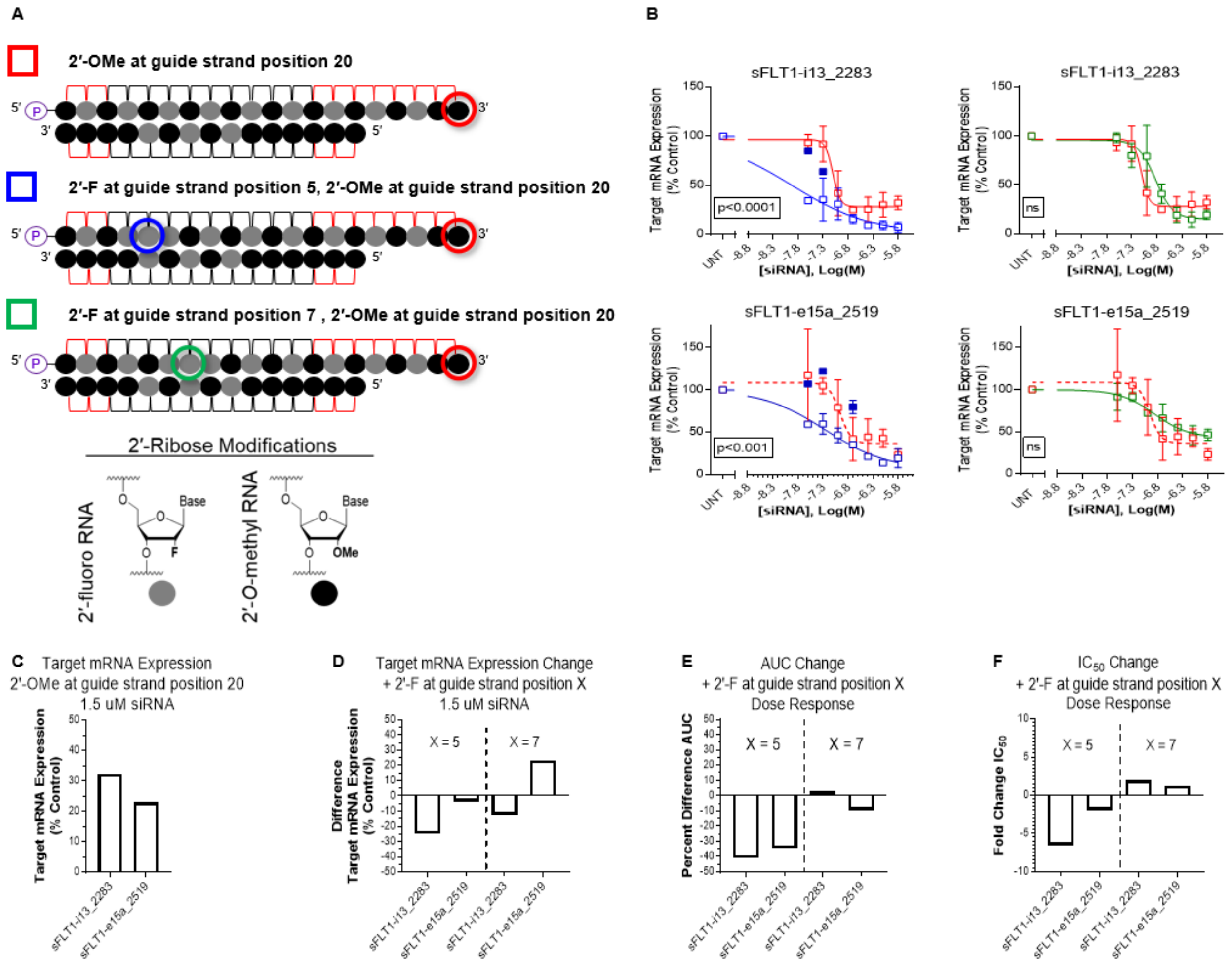

Figure S2. 2′-fluoro at position 5 but not 7 of 20-mer guide strands increases activity of fully modified, asymmetric siRNAs with 2′-*O*-methyl at 3′ guide strand termini. (A) Schematic representations of chemical modification scaffolds used; symbols next to each schematic are used in graphs shown in (B). Legend shows corresponding chemical structures for 2′-ribose modifications. (B) Dose response results (n=3, mean ± SD). Target name and start site of target sequence indicated in each graph. HeLa cells were treated with siRNAs at concentrations shown for 72 hours. mRNA levels were measured using the Dual-Glo® Luciferase Assay System and calculated as a percent of those from untreated cell controls. Statistical outliers are excluded from analysis, but are shown in the graphs as solid data points. Non-linear regression curves with $R^2<0.8$ are displayed as dashed rather than solid lines. p-values displayed on each graph calculated by two-way ANOVA. (C) Target mRNA expression with 1.5 μM of each siRNA with 2′-OMe at guide strand position 20. (D) Differences in target mRNA expression with 1.5 μM of each siRNA when 2′-F replaces 2′-OMe at guide strand position 5 or 7. (E) Percent differences in AUCs. (F) Fold changes in IC_{50} values, calculated by dividing the value obtained from siRNAs with 2′-F at guide strand position 5 or 7 by the value obtained from siRNAs with 2′-F at guide strand position 5 or 7; if this number was <1 the negative reciprocal is shown. (D, E, F) Positive or negative values indicate increases or decreases in values when 2′-F replaces 2′-OMe at guide strand position 5 or 7.

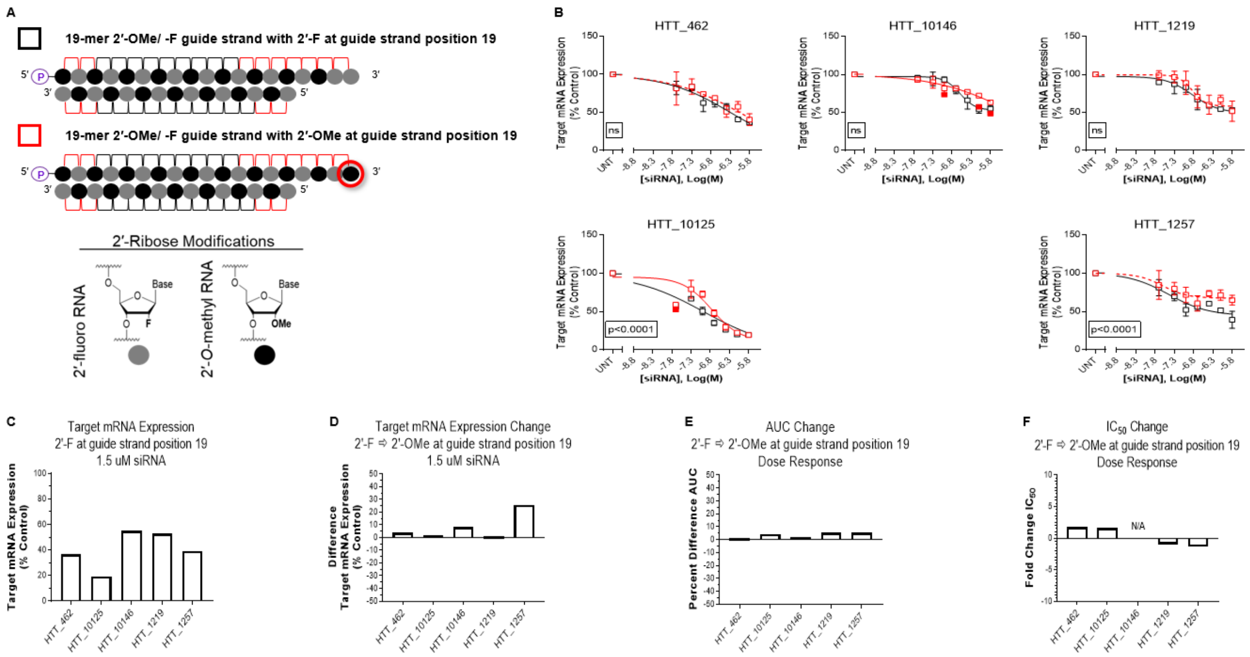

Figure S3. 2′-*O*-methyl at 3′ termini of 19-mer guide strands does not impact activity of fully modified, asymmetric siRNAs. (A) Schematic representations of chemical modification scaffolds used; symbols next to each schematic are used in graphs shown in (B). Legend shows corresponding chemical structures for 2′-ribose modifications. (B) Dose response results (n=3, mean ± SD). Target name and start site of target sequence indicated in each graph. HeLa cells were treated with siRNAs at concentrations shown for 72 hours. mRNA levels were measured using the Quantigene 2.0 RNA Assay and calculated as a percent of those from untreated cell controls. Statistical outliers were excluded from analyses, but are shown in the graphs as solid data points. Non-linear regression curves with $R^2<0.8$ are displayed as dashed rather than solid lines. p-values displayed on each graph calculated by two-way ANOVA. (C) Target mRNA expression with 1.5 µM of each siRNA with 2′-F at guide strand position 19. (D) Differences in target mRNA expression with 1.5 µM of each siRNA when 2′-OMe replaces 2′-F at guide strand position 19. (E) Percent differences in AUCs. (F) Fold changes in IC_{50} values, calculated by dividing the value obtained from siRNAs with 2′-OMe at guide strand position 19 by the value obtained from siRNAs with 2′-F at guide strand position 19; if this number was <1 the negative reciprocal is shown. "N/A" is shown if the Prism calculated IC_{50} value for one or both siRNAs for that sequence is greater than the top treatment dose (i.e. 1.5 µM). (D, E, F) Positive or negative values indicate increases or decreases in values when 2′-OMe replaces 2′-F at guide strand position 19.

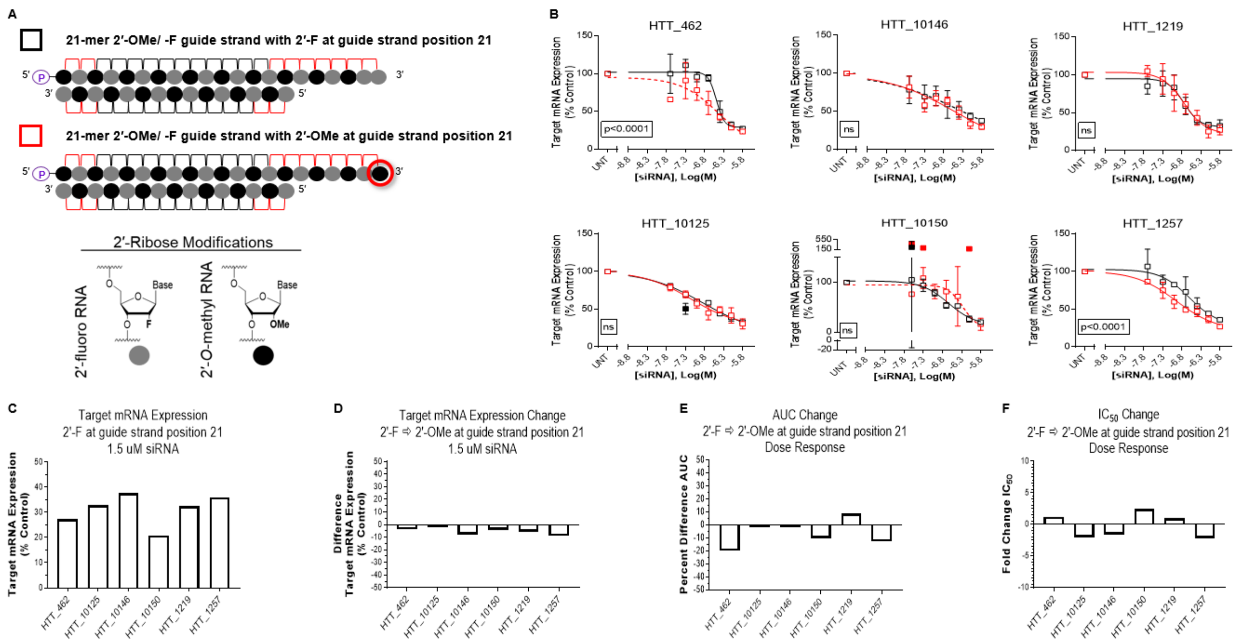

Figure S4. 2′-*O*-methyl at 3′ termini of 21-mer guide strands does not impact activity of fully modified, asymmetric siRNAs. (A) Schematic representations of chemical modification scaffolds used; symbols next to each schematic are used in graphs shown in (B). Legend shows corresponding chemical structures for 2′-ribose modifications. (B) Dose response results (n=3, mean ± SD). Target name and start site of target sequence indicated in each graph. HeLa cells were treated with siRNAs at concentrations shown for 72 hours. mRNA levels were measured using the Quantigene 2.0 RNA Assay and calculated as a percent of those from untreated cell controls. Statistical outliers were excluded from analyses, but are shown in the graphs as solid data points. Non-linear regression curves with $R^2<0.8$ are displayed as dashed rather than solid lines. p-values displayed on each graph calculated by two-way ANOVA. (C) Target mRNA expression with 1.5 μM of each siRNA with 2′-F at guide strand position 21. (D) Differences in target mRNA expression with 1.5 μM of each siRNA when 2′-OMe replaces 2′-F at guide strand position 21. (E) Percent differences in AUCs. (F) Fold changes in IC_{50} values, calculated by dividing the value obtained from siRNAs with 2′-OMe at guide strand position 21 by the value obtained from siRNAs with 2′-F at guide strand position 21; if this number was <1 the negative reciprocal is shown. (D, E, F) Positive or negative values indicate increases or decreases in values when 2′-OMe replaces 2′-F at guide strand position 21.

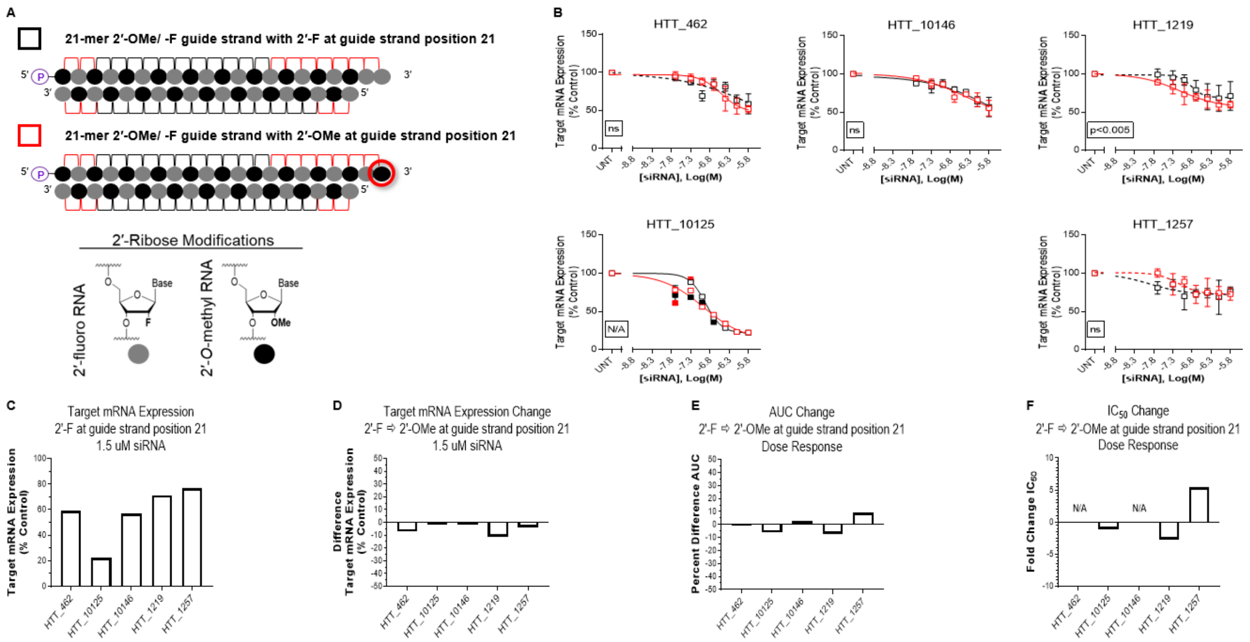

Figure S5. Sequences tested do not tolerate change from asymmetric to more symmetric 21-/19-mer siRNA structure. (A) Schematic representations of chemical modification scaffolds used; symbols next to each schematic are used in graphs shown in (B). Legend shows corresponding chemical structures for 2′-ribose modifications. (B) Dose response results (n=3, mean ± SD). Target name and start site of target sequence indicated in each graph. HeLa cells were treated with siRNAs at concentrations shown for 72 hours. mRNA levels were measured using the Quantigene 2.0 RNA Assay and calculated as a percent of those from untreated cell controls. Statistical outliers were excluded from analyses, but are shown in the graphs as solid data points. Non-linear regression curves with $R^2<0.8$ are displayed as dashed rather than solid lines. p-values displayed on each graph calculated by two-way ANOVA. Note that a p-value could not be calculated for HTT_10125 because all 3 points at the lowest test concentration for HTT_10125 siRNA with 3′-end 2′-F were excluded as statistical outliers. (C) Target mRNA expression with 1.5 µM of each siRNA with 2′-F at guide strand position 21. (D) Differences in target mRNA expression with 1.5 µM of each siRNA when 2′-OMe replaces 2′-F at guide strand position 21. (E) Percent differences in AUCs. (F) Fold changes in IC_{50} values, calculated by dividing the value obtained from siRNAs with 2′-OMe at guide strand position 21 by the value obtained from siRNAs with 2′-F at guide strand position 21; if this number was <1 the negative reciprocal is shown. “N/A” is shown if the Prism calculated IC_{50} value for one or both siRNAs for that sequence is greater than the top treatment dose (i.e. 1.5 µM). (D, E, F) Positive or negative values indicate increases or decreases in values when 2′-OMe replaces 2′-F at guide strand position 21.

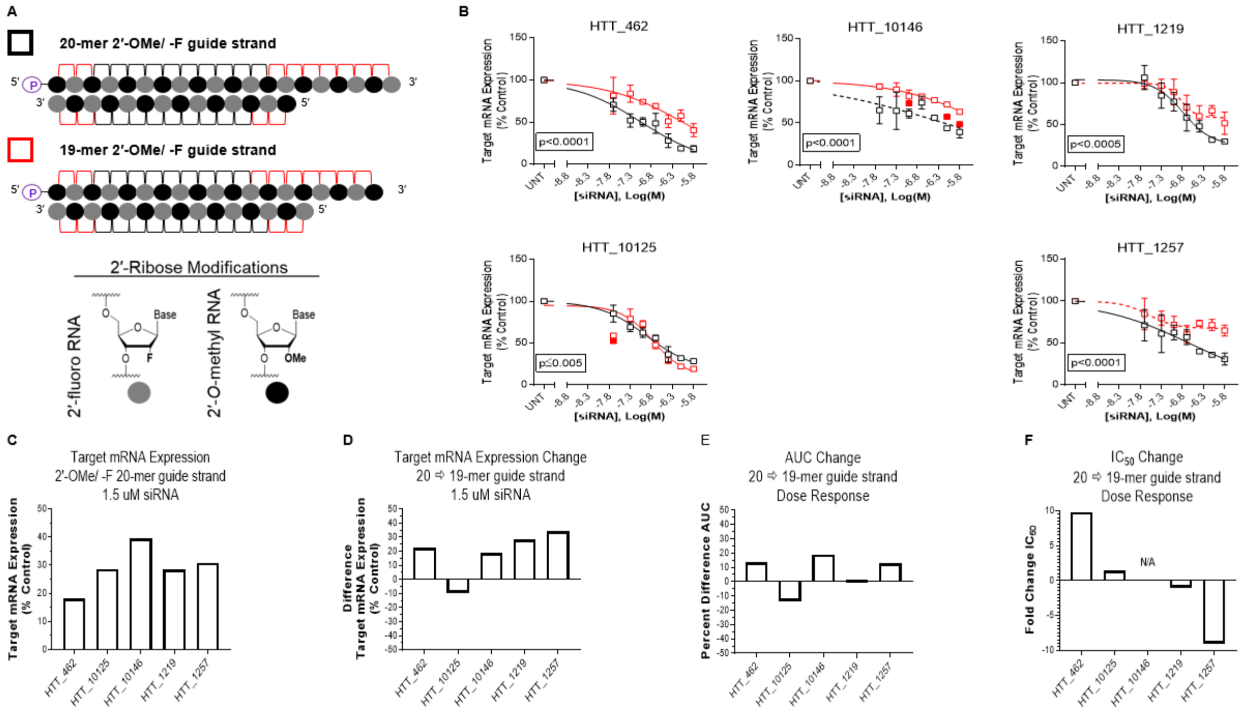

Figure S6. Sequences tested tolerate 20-/15-mer but not 19-/15-mer asymmetric siRNA structure. (A) Schematic representations of chemical modification scaffolds used; symbols next to each schematic are used in graphs shown in (B). Legend shows corresponding chemical structures for 2′-ribose modifications. (B) Dose response results (n=3, mean ± SD). Target name and start site of target sequence indicated in each graph. HeLa cells were treated with siRNAs at concentrations shown for 72 hours. mRNA levels were measured using the Quantigene 2.0 RNA Assay and calculated as a percent of those from untreated cell controls. Statistical outliers were excluded from analyses, but are shown in the graphs as solid data points. Non-linear regression curves with $R^2<0.8$ are displayed as dashed rather than solid lines. p-values displayed on each graph calculated by two-way ANOVA. (C) Target mRNA expression with 1.5 µM of each siRNA with a 20-mer guide strand and 2′-F at guide strand position 20. (D) Differences in target mRNA expression with 1.5 µM of each siRNA when 1 nucleotide is removed from the 3′ end of the guide strand. (E) Percent differences in AUCs. (F) Fold changes in IC_{50} values, calculated by dividing the value obtained from siRNAs with 19-mer guide strands by the value obtained from siRNAs with 20-mer guide strands; if this number was <1 the negative reciprocal is shown. "N/A" is shown if the Prism calculated IC_{50} value for one or both siRNAs for that sequence is greater than the top treatment dose (i.e. 1.5 µM). (D, E, F) Positive or negative values indicate increases or decreases in values when 1 nucleotide is removed from the 3′ end of the guide strand.

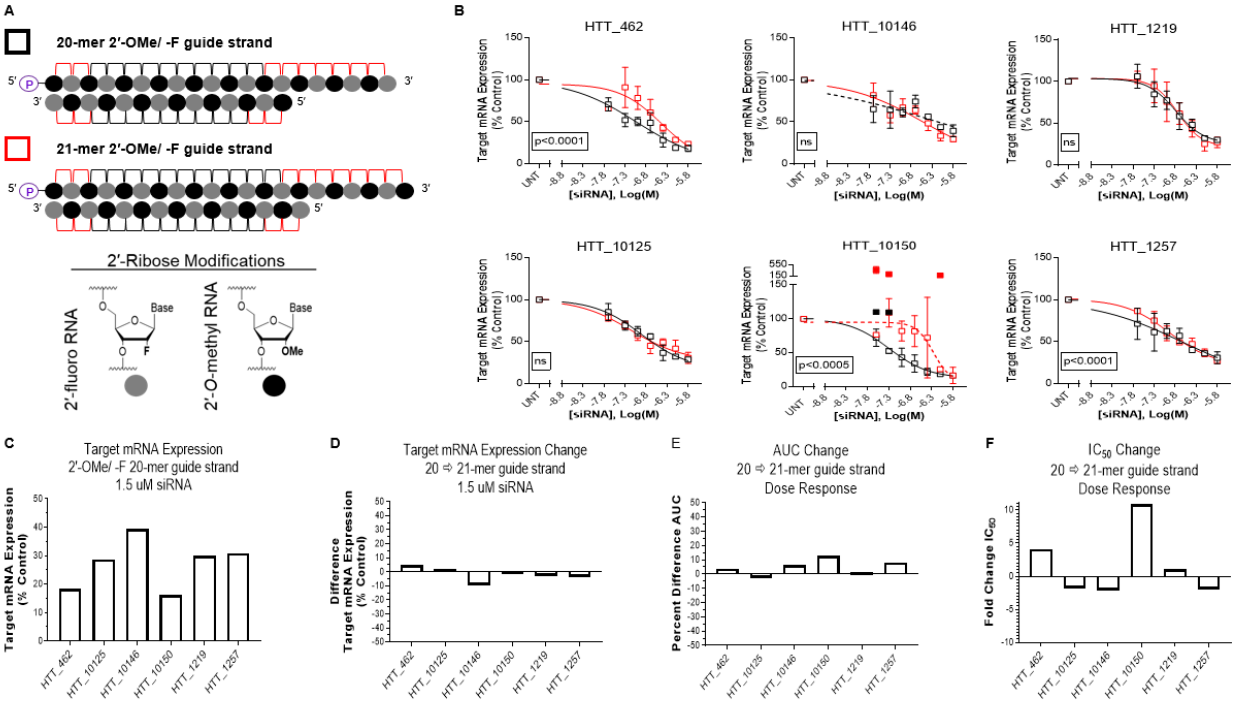

Figure S7. Sequences tested tolerate 20-/15-mer and 21-/15-mer asymmetric siRNA structure. (A) Schematic representations of chemical modification scaffolds used; symbols next to each schematic are used in graphs shown in (B). Legend shows corresponding chemical structures for 2′-ribose modifications. (B) Dose response results (n=3, mean ± SD). Target name and start site of target sequence indicated in each graph. HeLa cells were treated with siRNAs at concentrations shown for 72 hours. mRNA levels were measured using the Quantigene 2.0 RNA Assay and calculated as a percent of those from untreated cell controls. Statistical outliers were excluded from analyses, but are shown in the graphs as solid data points. Non-linear regression curves with $R^2<0.8$ are displayed as dashed rather than solid lines. p-values displayed on each graph calculated by two-way ANOVA. (C) Target mRNA expression with 1.5 µM of each siRNA with a 20-mer guide strand and 2′-F at guide strand position 20. (D) Differences in target mRNA expression with 1.5 µM of each siRNA when 1 nucleotide is added to the 3′ end of the guide strand. (E) Percent differences in AUCs. (F) Fold changes in IC_{50} values, calculated by dividing the value obtained from siRNAs with 21-mer guide strands by the value obtained from siRNAs with 20-mer guide strands; if this number was <1 the negative reciprocal is shown. (D, E, F) Positive or negative values indicate increases or decreases in values when 1 nucleotide is added to the 3′ end of the guide strand.

Supplemental Tables

Table S1. Values from Figure S1. When looking at differences, positive values indicate an increase in values with the application of the indicated modification change. IC_{50} fold change was calculated by dividing the value obtained with the indicated modification change by the value obtained without it. However, if this number was <1 the negative reciprocal is listed (e.g. 0.75, or a drop of 25% from the original value is reported as -1.3 fold change).

**Guide Strand Modification Pattern**	**2′-OMe/ -F**	**2′-OMe at Position 20**	**+ 2′-OMe at Position 20**
**Target**	**% Target mRNA Expression with 1.5 uM siRNA**		**Difference % Target mRNA Expression with 1.5 uM siRNA**
sFLT1-i13_2283	18.9	32.3	13.4
**Target**	**Area Under Curve**		**% Difference Area Under Curve**
sFLT1-i13_2283	622.4	760.0	19.9
**Target**	**IC_{50} Value (nM)**		**Fold Change IC_{50} Value**
sFLT1-i13_2283	204.1	292.8	1.4

Table S2. Values from Figure S2. When looking at differences, positive or negative values indicate an increase or decrease in values with the application of the indicated modification change. IC_{50} fold change was calculated by dividing the value obtained with the indicated modification change by the value obtained without it. However, if this number was <1 the negative reciprocal is listed (e.g. 0.75, or a drop of 25% from the original value is reported as -1.3 fold change).

**Guide Strand Modification Pattern**	**2′-OMe at Position 20**	**2′-OMe at Position 20 and 2′-F at Position 5**	**2′-OMe at Position 20 and 2′-F at Position 7**	**+ 2′-F at Position 5**	**+ 2′-F at Position 7**
**Target**	**% Target mRNA Expression with 1.5 uM siRNA**			**Difference % Target mRNA Expression with 1.5 uM siRNA**	
sFLT1-i13_2283	32.3	7.3	19.7	-25.0	-12.7
sFLT1-e15a_2519	23.0	19.0	46.3	-4.0	23.3
**Target**	**Area Under Curve**			**% Difference Area Under Curve**	
sFLT1-i13_2283	605.4	399.6	623.5	-41.0	2.9
sFLT1-e15a_2519	700.5	493.6	635.5	-34.7	-9.7
**Target**	**IC_{50} Value (nM)**			**Fold Change IC_{50} Value**	
sFLT1-i13_2283	78.7	12.0	149.7	-6.6	1.9
sFLT1-e15a_2519	110.1 [a]	55.5	135.5	-2.0	1.2

[a]The R^2 value for the fitted curve used to calculate the IC_{50} value <0.8.

Table S3. Values from Figure S3. When looking at differences, positive or negative values indicate an increase or decrease in values with the application of the indicated modification change. IC_{50} fold change was calculated by dividing the value obtained with the indicated modification change by the value obtained without it. However, if this number was <1 the negative reciprocal is listed (e.g. 0.75, or a drop of 25% from the original value is reported as -1.3 fold change). "N/A" = not applicable.

**Guide Strand Modification Pattern**	**2′-F at Position 19**	**2′-OMe at Position 19**	**+ 2′-OMe at Position 19**
**Target**	**% Target mRNA Expression with 1.5 µM siRNA**		**Difference % Target mRNA Expression with 1.5 uM siRNA**
HTT_462	36.6	40.6	4.0
HTT_10125	19.4	19.3	-0.2
HTT_10146	55.1	58.5	3.4
HTT_1219	52.7	51.8	-0.9
HTT_1257	39.2	65.0	25.8
**Target**	**Area Under Curve**		**% Difference Area Under Curve**
HTT_462	600.6	608.3	1.3
HTT_10125	493.5	515.6	4.4
HTT_10146	666.3	665.8	-0.1
HTT_1219	638.1	674.4	5.5
HTT_1257	592.9	627.2	5.6
**Target**	**IC_{50} Value (nM)**		**Fold Change IC_{50} Value**
HTT_462	506.7	893.8[a]	1.8
HTT_10125	106.9	171.3	1.6
HTT_10146	252.8	>1500[b]	N/A
HTT_1219	146.8	146.7[a]	-1.0
HTT_1257	35.61	25.01[a]	-1.4

[a]The R^2 value for the fitted curve used to calculate the IC_{50} value <0.8.
[b]The Prism calculated IC_{50} value is greater than the top treatment dose (i.e. 1.5 µM) and is therefore excluded from analysis (see Materials & Methods).

Table S4. Dose response data at top three treatment concentrations for HTT_10125 with 20- and 19-mer guide strands. When looking at differences, positive or negative values indicate an increase or decrease in values with the application of the indicated modification change.

<table>
<tr><th></th><th>Guide Strand Modification Pattern</th><th>2′-F at 3′ end of Guide Strand</th><th>2′-OMe at 3′ end of Guide Strand</th><th colspan="2">+ 2′-OMe at 3′ end of Guide Strand</th></tr>
<tr><th>siRNA</th><th>Dose (μM)</th><th colspan="2">% Target mRNA Expression</th><th>Difference % Target mRNA Expression</th><th>Average Difference % Target mRNA Expression</th></tr>
<tr><td rowspan="3">HTT_10125 with 20-mer Guide Strand</td><td>1.5</td><td>28.7</td><td>33.4</td><td>4.7</td><td rowspan="3">7.5</td></tr>
<tr><td>0.75</td><td>32.2</td><td>39.5</td><td>7.3</td></tr>
<tr><td>0.375</td><td>36.7</td><td>47.3</td><td>10.6</td></tr>
<tr><td rowspan="3">HTT_10125 with 19-mer Guide Strand</td><td>1.5</td><td>19.4</td><td>19.3</td><td>-0.2</td><td rowspan="3">1.8</td></tr>
<tr><td>0.75</td><td>20.0</td><td>22.3</td><td>2.3</td></tr>
<tr><td>0.375</td><td>26.3</td><td>29.7</td><td>3.4</td></tr>
</table>

Table S5. Values from Figure S4. When looking at differences, positive or negative values indicate an increase or decrease in values with the application of the indicated modification change. IC_{50} fold change was calculated by dividing the value obtained with the indicated modification change by the value obtained without it. However, if this number was <1 the negative reciprocal is listed (e.g. 0.75, or a drop of 25% from the original value is reported as -1.3 fold change).

**Guide Strand Modification Pattern**	**2′-F at Position 21**	**2′-OMe at Position 21**	**+ 2′-OMe at Position 21**
**Target**	**% Target mRNA Expression with 1.5 uM siRNA**		**Difference % Target mRNA Expression with 1.5 uM siRNA**
HTT_462	27.4	23.4	-4.0
HTT_10125	32.8	30.7	-2.1
HTT_10146	37.7	29.7	-8.0
HTT_10150	20.9	16.4	-4.5
HTT_1219	32.6	27.0	-5.6
HTT_1257	36.0	27.0	-9.0
**Target**	**Area Under Curve**		**% Difference Area Under Curve**
HTT_462	674.4	551.6	-20.0
HTT_10125	586.0	576.9	-1.6
HTT_10146	585.6	587.3	0.3
HTT_10150	665.4	601.6	-10.1
HTT_1219	608.1	663.3	8.7
HTT_1257	681.4	599.8	-12.7
**Target**	**IC_{50} Value (nM)**		**Fold Change IC_{50} Value**
HTT_462	321.8	372.9[a]	1.2
HTT_10125	149.1	70.2	-2.1
HTT_10146	489.5[a]	310.5	-1.6
HTT_10150	228.5	546.1[a]	2.4
HTT_1219	178.8	181.1	1.0
HTT_1257	248.6	113.6	-2.2

[a]The R^2 value for the fitted curve used to calculate the IC_{50} value <0.8.

Table S6. Values from Figure S5. When looking at differences, positive or negative values indicate an increase or decrease in values with the application of the indicated modification change. IC_{50} fold change was calculated by dividing the value obtained with the indicated modification change by the value obtained without it. However, if this number was <1 the negative reciprocal is listed (e.g. 0.75, or a drop of 25% from the original value is reported as -1.3 fold change). “N/A” = not applicable.

**Guide Strand Modification Pattern**	**2′-F at Position 21**	**2′-OMe at Position 21**	**+ 2′-OMe at Position 21**
**Target**	**% Target mRNA Expression with 1.5 uM siRNA**		**Difference % Target mRNA Expression with 1.5 uM siRNA**
HTT_462	59.0	51.9	-7.1
HTT_10125	22.3	22.6	0.3
HTT_10146	56.8	55.2	-1.6
HTT_1219	71.2	59.8	-11.4
HTT_1257	76.5	72.9	-3.6
**Target**	**Area Under Curve**		**% Difference Area Under Curve**
HTT_462	660.7	666.6	0.9
HTT_10125	601.9	565.8	-6.2
HTT_10146	644.6	661.9	2.6
HTT_1219	684.4	637.7	-7.1
HTT_1257	622.7	684.4	9.4
**Target**	**IC_{50} Value (nM)**		**Fold Change IC_{50} Value**
HTT_462	>1500[a, b]	309.3	N/A
HTT_10125	121.9	109.1	-1.1
HTT_10146	>1500[b]	424.2	N/A
HTT_1219	151.3[a]	56.2	-2.7
HTT_1257	12.87[a]	69.6[a]	5.4

[a]The R^2 value for the fitted curve used to calculate the IC_{50} value <0.8.
[b]The Prism calculated IC_{50} value is greater than the top treatment dose (i.e. 1.5 μM) and is therefore excluded from analysis (see Materials & Methods).

Table S7. Values from Figure S6. When looking at differences, positive or negative values indicate an increase or decrease in values with the application of the indicated modification change. IC_{50} fold change was calculated by dividing the value obtained with the indicated modification change by the value obtained without it. However, if this number was <1 the negative reciprocal is listed (e.g. 0.75, or a drop of 25% from the original value is reported as -1.3 fold change). "N/A" = not applicable.

**Guide Strand Modification Pattern**	**2′-OMe/ -F 20-mer**	**2′-OMe/ -F 19-mer**	**- 1 Nucleotide**
**Target**	**% Target mRNA Expression with 1.5 uM siRNA**		**Difference % Target mRNA Expression with 1.5 uM siRNA**
HTT_462	18.3	40.6	22.3
HTT_10125	28.7	19.3	-9.4
HTT_10146	39.5	58.5	19.0
HTT_1219	30.1	58.5	28.4
HTT_1257	30.9	65.0	34.1
**Target**	**Area Under Curve**		**% Difference Area Under Curve**
HTT_462	531.1	608.3	13.6
HTT_10125	592.9	515.6	-13.9
HTT_10146	550.2	665.8	19.0
HTT_1219	666.7	674.4	1.1
HTT_1257	553.0	627.2	12.6
**Target**	**IC_{50} Value (nM)**		**Fold Change IC_{50} Value**
HTT_462	91.6	893.8	9.8
HTT_10125	122.9	171.3	1.4
HTT_10146	643.3[a]	>1500[b]	N/A
HTT_1219	164.0	146.7[a]	-1.1
HTT_1257	227.3	25.0[a]	-9.1

[a]The R^2 value for the fitted curve used to calculate the IC_{50} value <0.8.
[b]The Prism calculated IC_{50} value is greater than the top treatment dose (i.e. 1.5 μM) and is therefore excluded from analysis (see Materials & Methods).

Table S8. Values from Figure S7. When looking at differences, positive or negative values indicate an increase or decrease in values with the application of the indicated modification change. IC_{50} fold change was calculated by dividing the value obtained with the indicated modification change by the value obtained without it. However, if this number was <1 the negative reciprocal is listed (e.g. 0.75, or a drop of 25% from the original value is reported as -1.3 fold change).

**Guide Strand Modification Pattern**	**2′-OMe/ -F 20-mer**	**2′-OMe/ -F 21-mer**	**+ 1 Nucleotide**
**Target**	**% Target mRNA Expression with 1.5 uM siRNA**		**Difference % Target mRNA Expression with 1.5 uM siRNA**
HTT_462	18.3	23.4	5.1
HTT_10125	28.7	30.7	2.0
HTT_10146	39.5	29.7	-9.7
HTT_10150	16.2	16.4	0.1
HTT_1219	30.1	27.0	-3.1
HTT_1257	30.9	27.0	-3.9
**Target**	**Area Under Curve**		**% Difference Area Under Curve**
HTT_462	531.1	551.6	3.8
HTT_10125	592.9	576.9	-2.7
HTT_10146	550.2	587.3	6.5
HTT_10150	528.8	601.6	12.9
HTT_1219	666.7	663.3	-0.5
HTT_1257	553.0	599.8	8.1
**Target**	**IC_{50} Value (nM)**		**Fold Change IC_{50} Value**
HTT_462	91.61	372.9	4.1
HTT_10125	122.9	70.2	-1.8
HTT_10146	643.3[a]	310.5	-2.1
HTT_10150	50.3	546.1[a]	10.9
HTT_1219	164.0	181.0	1.1
HTT_1257	227.3	113.6	-2.0

[a]The R^2 value for the fitted curve used to calculate the IC_{50} value <0.8.

Table S9. Oligonucleotides examined in PyMol. 'P' denotes phosphate; 'VP-T' denotes 2′-O-MOE-thymidine-(E)-5′-vinylphosphonate; '#' denotes phosphorothioate instead of phosphodiester; 'm' denotes 2′-O-methyl; 'f' denotes 2′-fluoro; 'me' denotes 2′-methoxyethyl; 'A' denotes Adenine; 'U' denotes Uracil; 'G' denotes Guanosine; 'C' denotes Cysteine.

**PDB Reference Number**	**Guide Strand Sequence with Chemical Modification Pattern**
4W5N, 4W5Q	P(U)(U)(C)(A)(C)(A)(U)(U)(G)(C)(C)(C)(A)(A)(G)(U)(C)(U)(C)(U)(U)
5JS2	VP-T#(fU)#(mA)(fU)#(mC)(fU)#(mA)(fU)#(mA)(fA)#(mU)(fG)#(mA)(fU)#(mC)#(fA)#(mG)#(fG)#(mU)#(meA)#(meA)
3MJ0	(C)(G)(U)(U)(A)(C)(G)(C)(mU)

Table S10. Oligonucleotide duplexes used in in vitro efficacy and thermal melt studies. Information for each duplex is boxed with a thick outside border. 'P' denotes phosphate; '#' denotes phosphorothioate instead of phosphodiester; 'm' denotes 2′-O-methyl; 'f' denotes 2′-fluoro; 'r' denotes 2′-OH; 'A' denotes Adenine; 'U' denotes Uracil; 'G' denotes Guanosine; 'C' denotes Cysteine; 'TegChol' denotes Triethylene Glycol linker + Cholesterol conjugate.
