# Supplementary material for: 2′-O-Methyl at 20-mer Guide Strand 3′ Termini May Negatively Affect Target Silencing Activity of Fully Chemically Modified siRNA
Source: Mol Ther Nucleic Acids. 2020 May 15;21:266–77. doi: 10.1016/j.omtn.2020.05.010 (PMC7327867; doi:10.1016/j.omtn.2020.05.010)
Supplement: Document S2. Article plus Supplemental Information [file mmc3.pdf]

Molecular Therapy
Nucleic Acids
Original Article

2′-*O*-Methyl at 20-mer Guide Strand 3′ Termini May Negatively Affect Target Silencing Activity of Fully Chemically Modified siRNA

Sarah M. Davis,[1] Jacquelyn Sousa,[1] Lorenc Vangjeli,[1] Matthew R. Hassler,[1] Dimas Echeverria,[1] Emily Knox,[1] Anton A. Turanov,[1] Julia F. Alterman,[1] and Anastasia Khvorova[1,2]

[1]RNA Therapeutics Institute, University of Massachusetts Medical School, Worcester, MA, USA; [2]Department of Molecular Medicine, University of Massachusetts Medical School, Worcester, MA 01605, USA

Small interfering RNAs (siRNAs) have the potential to treat a broad range of diseases. siRNAs need to be extensively chemically modified to improve their bioavailability, safety, and stability *in vivo*. However, chemical modifications variably impact target silencing for different siRNA sequences, making the activity of chemically modified siRNA difficult to predict. Here, we systematically evaluated the impact of 3′ terminal modifications (2′-*O*-methyl versus 2′-fluoro) on guide strands of different length and showed that 3′ terminal 2′-*O*-methyl modification negatively impacts activity for >60% of siRNA sequences tested but only in the context of 20- and not 19- or 21-nt-long guide strands. These results indicate that sequence, modification pattern, and structure may cooperatively affect target silencing. Interestingly, the introduction of an extra 2′-fluoro modification in the seed region at guide strand position 5, but not 7, may partially compensate for the negative impact of 3′ terminal 2′-*O*-methyl modification. Molecular modeling analysis suggests that 2′-*O*-methyl modification may impair guide strand interactions within the PAZ domain of argonaute-2, which may affect target recognition and cleavage, specifically when guide strands are 20-nt long. Our findings emphasize the complex nature of modified RNA-protein interactions and contribute to design principles for chemically modified siRNAs.

INTRODUCTION

Small interfering RNA (siRNA) interacts with the RNA-induced silencing complex (RISC) to degrade complementary mRNA and prevent protein translation, making it a powerful tool for silencing disease-causing genes.[1] Extensive chemical modification improves the stability of siRNAs *in vivo*, which is especially important for conjugated siRNAs to achieve long-term target silencing.[2]

A common siRNA modification site is the 2′ position of ribose, where 2′-*O*-methyl (2′-OMe) and 2′-fluoro (2′-F) are frequently used. The majority of clinical-stage compounds is fully modified and possess a larger fraction of 2′-OMe modifications than 2′-F modifications because the former is more stabilizing against nucleases.[3] Indeed, an increase of 2′-OMe content enhances siRNA potency and duration of effect *in vivo*.[4] However, the inclusion of 2′-OMe at certain positions can negatively impact activity—e.g., guide strand position 14 does not tolerate 2′-OMe.[4–6]

Whereas chemical modifications are essential for conjugated siRNA efficacy *in vivo*, they may negatively impact one or more steps of RISC function, including the following: when (1) the siRNA duplex is loaded into RISC, and the passenger strand is released; (2) the mRNA target is recognized by guide strand-loaded RISC; (3) the siRNA-mRNA target adopts the optimal configuration for cleavage; (4) the siRNA-mRNA target undergoes endonucleolytic cleavage; and (5) the product is released. However, the individual and combined effects of different types of modifications, like 2′-OMe and 2′-F on RISC function, are not well understood.

Many siRNA sequences that are active when unmodified lose activity when they are extensively chemically modified.[5] Moreover, chemical modification patterns variably impact the efficacy of different sequences. Therefore, the screening of multiple chemical variants for each siRNA sequence is currently required to generate compounds with optimal stability and activity. A better understanding of general chemical modification rules (i.e., those applicable to many different sequences) may reduce the amount of effort required to perform these extensive screens.

Here, we demonstrate that 3′ terminal 2′-OMe modification of 20- but not 19- or 21-mer guide strands may reduce the activity of fully modified, asymmetric siRNAs and that this negative effect may be partially compensated by including an additional 2′-F modification at guide strand position 5. Preliminary structural modeling provides a potential explanation for these results and emphasizes the

Received 2 April 2020; accepted 12 May 2020;
https://doi.org/10.1016/j.omtn.2020.05.010.

Correspondence: Anastasia Khvorova, PhD, RNA Therapeutics Institute, University of Massachusetts Medical School, AS4-1049, 368 Plantation Street, Worcester, MA 01605, USA.
E-mail: anastasia.khvorova@umassmed.edu

 © 2020 The Authors.
This is an open access article under the CC BY license (http://creativecommons.org/licenses/by/4.0/).

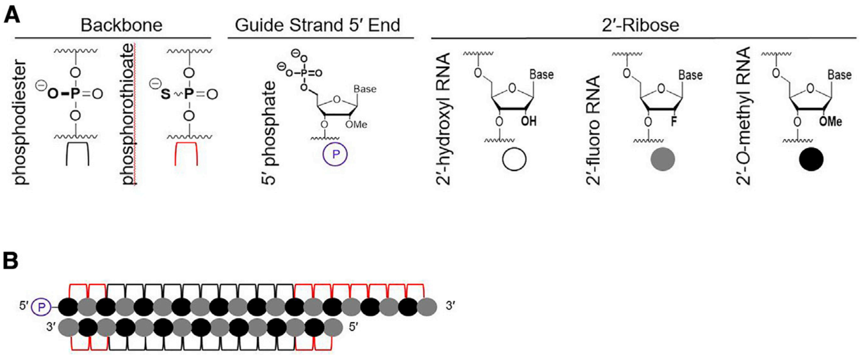

Figure 1. siRNA Chemical Scaffold Used in this Study

(A) Legend showing corresponding chemical structures for chemistries used in this study. (B) Schematic representation of asymmetric siRNA with an alternating 2′-OMe/-F chemical pattern.

complexity and dynamic nature of chemically modified siRNA-RISC interactions.

RESULTS

2′-OMe at the 3′ Termini of 20-mer Guide Strands Negatively Impacts Target Silencing for Fully Modified, Asymmetric siRNAs

To evaluate the impact of position-specific chemical modification changes, we utilized an asymmetric siRNA, fully chemically modified with alternating 2′-OMe and 2′-F groups (i.e., the standard modification pattern described by Allerson et al.[7]) (Figure 1). In this configuration, a 20-mer guide strand is paired to a 15-mer passenger strand, creating a single-stranded phosphorothioate (PS) tail on the 3′ end of the guide strand that contributes to cellular uptake, likely similarly to the mechanism used by antisense oligonucleotides.[8,9] When conjugated to cholesterol, these compounds are efficiently internalized by all cell types via early endosome antigen-1 (EEA1)-associated endocytosis[10] and result in productive silencing *in vitro* and *in vivo*,[2] which simplifies logistics for *in vitro* screens to evaluate siRNA efficacy.

We observed that incorporating 2′-OMe at 3′ guide strand termini negatively impacted siRNA activity for some sequences. To investigate this phenomenon further and systematically, we synthesized a panel of 16 siRNAs targeting eight different sequences within three genes; the two siRNAs for each sequence differed only in the type of modification (i.e., 2′-OMe versus 2′-F) on the 3′ end of their guide strand (Figure 2). We treated HeLa cells with these siRNAs for 72 h and measured target mRNA expression levels using Dual-Glo Luciferase or Quantigene 2.0 RNA assay systems (see Materials and Methods). To ensure that any differences we identified were robust, each siRNA was evaluated at seven concentrations; these dose responses and a summary of results are shown in Figure 2 and Table 1.

The incorporation of the 2′-OMe modification at 3′ guide strand termini had a statistically significant impact on efficacy for five of the eight sequences tested (Figure 2B). Figure 2C plots target mRNA expression levels after treatment with the top dose (i.e., 1.5 μM) of each siRNA with 2′-F at guide strand position 20. Figure 2D plots the differences in target mRNA expression levels observed at the top dose when 2′-OMe replaces 2′-F at guide strand position 20. All siRNAs show decreased efficacy at this dose except for HTT_10146 and HTT_1219, for which target mRNA expression levels decreased by ≤1%. Figure 2E plots percent differences in the areas under the dose response curves (AUCs) in Figure 2B. For each test concentration of siRNA, if the replacement of 2′-F with 2′-OMe at 3′ guide strand termini increased target mRNA expression, then there is a positive shift along the y axis, which increases the AUC. Thus, a positive change in the AUC reflects an overall negative change in efficacy across all treatment concentrations. For all siRNAs except HTT_10125, AUCs increase. Positive changes in AUCs also reflect negative changes in potency, because if the dose required to achieve similar target mRNA expression levels increases, then there is a positive shift along the x axis. The half-maximal inhibitory concentration (IC_{50}) is also a valuable measure of potency when it can be calculated from a sigmoidal curve. Figure 2F plots fold changes in IC_{50} values. It shows that for all siRNAs except HTT_1257, the replacement of 2′-F with 2′-OMe at guide strand position 20 increases IC_{50} values by 1.3- to 7.3-fold. Interestingly, the most active sequences (i.e., those that demonstrate the highest amounts of silencing at the top treatment dose, lowest AUCs, and lowest IC_{50} values—sFLT1-i13_2283, sFLT1-e15a_2519, and HTT_10150) are the most negatively impacted by 2′-OMe at guide strand position 20 (Table 1; Figures 2C, 2E, and 2F). Similarly, the efficacies of the least effective sequences (i.e., HTT_10146, which demonstrates the lowest amount of target silencing at the top treatment dose, and HTT_10125 and HTT_1219, which have the highest AUCs) are the least negatively impacted, as reflected by low or negative percent differences in AUCs (Figure 2E) and nonsignificant p values (Figure 2B). This suggests that there are other parameters limiting the efficacies of these sequences that outweigh the contribution of the 3′ end guide strand modification. The replacement of 2′-F with 2′-OMe at guide strand position 20 also reduced the activity of sFLT1-i13_2283 siRNA with a 20-mer guide strand and 14-mer passenger strand (Figure S1; Table S1), indicating that the length of the single-stranded PS tail (i.e., 6 or 5) does not impact its negative effects.

These data show that replacing 2′-F with 2′-OMe at 3′ guide strand termini reduces the target silencing activity of siRNAs with guide strands that are 20 nt long and conform to the standard 2′-F/ 2′-OMe alternating pattern, described by Allerson et al.[7] Within the contexts of this guide strand length and chemical pattern, the negative impacts of this modification appear generally applicable to many different siRNA sequences. However, more active sequences demonstrated bigger reductions in activity, so we next investigated whether the negative contribution of 3′ guide strand terminal 2′-OMe could be alleviated for the three most active siRNAs—sFLT1-i13_2283, sFLT1-e15a_2519, and HTT_10150—by enhancing seed-based (i.e., guide strand positions 2–8) target recognition.

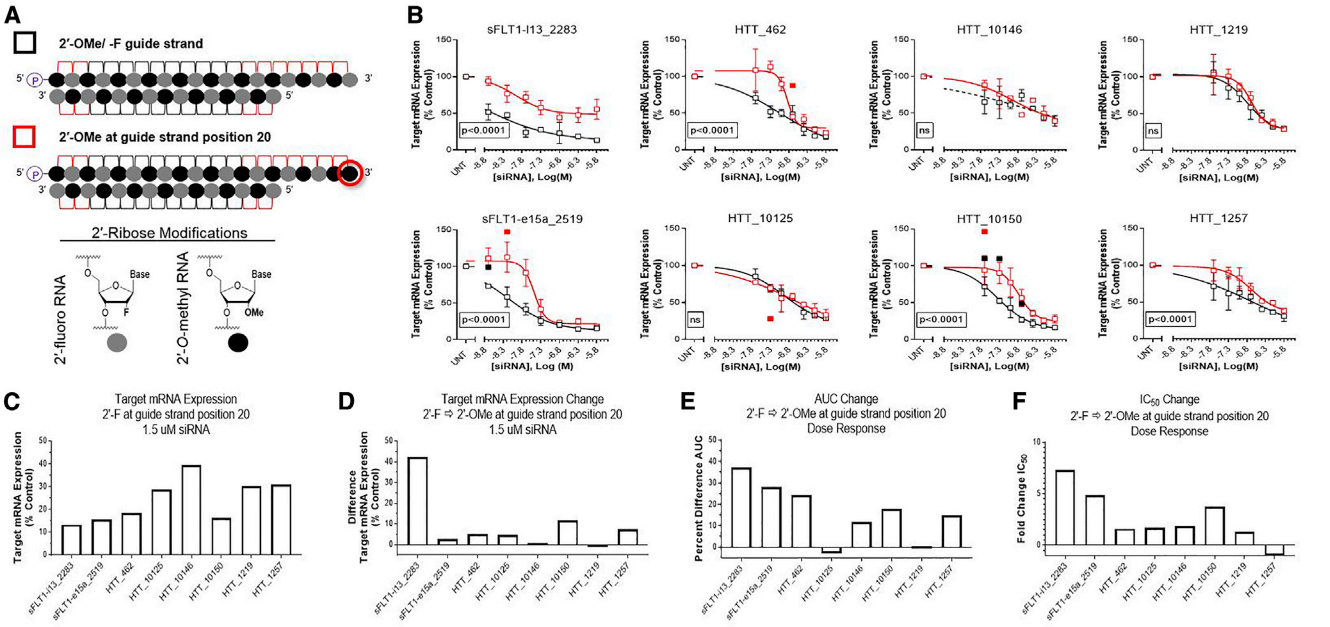

Figure 2. 2′-*O*-Methyl at 3′ Termini of 20-mer Guide Strands Decreases Activity of Fully Modified, Asymmetric siRNAs

(A) Schematic representations of chemical modification scaffolds used; symbols next to each schematic are used in graphs shown in (B). Legend shows corresponding chemical structures for 2′-ribose modifications. (B) Dose response results (n = 3, mean ± SD). Target name and start site of target sequence are indicated in each graph. HeLa cells were treated with siRNAs at concentrations shown for 72 h. mRNA levels were measured using the Dual-Glo Luciferase Assay System (*sFLT1-i13*, *sFLT1-e15a*) or Quantigene 2.0 RNA Assay (*HTT*) and calculated as a percent of those from untreated cell controls. Statistical outliers were excluded from analyses but are shown in the graphs as solid data points. Nonlinear regression curves with $R^2 < 0.8$ are displayed as dashed rather than solid lines. p values displayed on each graph were calculated by two-way ANOVA. (C) Target mRNA expression with 1.5 μM of each siRNA with 2′-F at guide strand position 20. (D) Differences in target mRNA expression with 1.5 μM of each siRNA when 2′-OMe replaces 2′-F at guide strand position 20. (E) Percent differences in AUCs. (F) Fold changes in IC_{50} values, calculated by dividing the value obtained from siRNAs with 2′-OMe at guide strand position 20 by the value obtained from siRNAs with 2′-F at guide strand position 20; if this number was <1, then the negative reciprocal is shown. (D–F) Positive or negative values indicate increases or decreases in values when 2′-OMe replaces 2′-F at guide strand position 20.

2′-F at Position 5 of 20-mer Guide Strands with 3′ Terminal 2′-OMe Improves Target Silencing for Fully Modified, Asymmetric siRNAs

2′-F modifications likely cause tighter base stacking than 2′-OMe modifications[11,12] and are therefore predicted to enhance target binding and in turn, improve siRNA activity. Thus, we replaced 2′-OMe with 2′-F at guide strand position 5 in the seed region (Figure 3A) and measured target mRNA expression levels to see if this modification change could improve the activity of siRNAs containing 3′ guide strand terminal 2′-OMe.

Figure 3 shows that incorporating 2′-F at position 5 had a compensatory effect on three different siRNAs, for which target silencing activities were significantly, negatively affected by 2′-OMe modification at 3′ guide strand termini. The summary of changes in efficacy and potency are shown in Table 1. This chemical modification enhanced target mRNA expression levels at the maximum treatment dose (1.5 μM siRNA) (Figure 3D) and over the full range of siRNA concentrations tested (Figure 3E) at significance levels <0.005 (Figure 3B) and also decreased IC_{50} values by 1.2- to 1.9-fold (Figure 3F). Increased activity was also demonstrated for sFLT1-i13_2283 and sFLT1-e15a_2519 siRNAs with passenger strands that deviated from the alternating 2′-F/2′-OMe chemical pattern by including 80% 2′-OMe modifications (Figure S2; Table S2), suggesting that the chemical pattern of the passenger strand does not impact the positive effects of this modification change.

The siRNA seed region corresponds to guide strand positions 2–8, so we next investigated if the enhancement in activity observed when 2′-OMe was replaced by 2′-F in the seed was specific to position 5. Interestingly, the incorporation of 2′-F at guide strand position 7 did not significantly improve activity for the siRNAs tested; in fact, we observed a minor negative impact (Figure S2; Table S2). These results suggest that conformational fitting within the seed, rather than target affinity within the seed, is the bigger contributing factor to changes in siRNA activity.

2′-F modifications indeed have slightly higher affinity to RNA compared to 2′-OMe,[13] but 2′-F is also smaller in size and thus, affords better base-stacking interactions,[11] which may increase duplex stability and/or enhance seed-based target scanning. To investigate whether these properties contributed to our observations, we measured the impact of these modifications at positions 5, 7, and 20 on the thermal melting temperatures (Tms) of sFLT1-i13_2283

Table 1. Values

Guide Strand Modification Pattern	2′-OMe/-F	2′-OMe at Position 20	2′-OMe at Position 20 and 2′-F at Position 5	+2′-OMe at Position 20	+2′-OMe at Position 20 and 2′-F at Position 5
Target	% Target mRNA Expression with 1.5 μM siRNA			Difference % Target mRNA Expression with 1.5 μM siRNA	
sFLT1-i13_2283	13.3	55.7	15.7	42.3	−40.0
sFLT1-e15a_2519	15.7	18.3	11.3	2.7	−7.0
HTT_462	18.3	23.5	N/D	5.1	N/D
HTT_10125	28.7	33.4	N/D	4.7	N/D
HTT_10146	39.5	39.1	N/D	−0.3	N/D
HTT_10150	16.2	27.8	18.3	11.6	−9.5
HTT_1219	30.1	28.8	N/D	−1.3	N/D
HTT_1257	30.9	38.4	N/D	7.5	N/D
Target	Area under Curve			% Difference Area under Curve	
sFLT1-i13_2283	410.3	597.0	486.1	37.1	−20.5
sFLT1-e15a_2519	471.1	626.3	566.4	28.3	−10.0
HTT_462	531.1	679.2	N/D	24.5	N/D
HTT_10125	592.9	575.2	N/D	−3.0	N/D
HTT_10146	550.2	620.2	N/D	12.0	N/D
HTT_10150	524.2	627.1	565.8	17.9	−10.3
HTT_1219	666.7	669.1	N/D	0.4	N/D
HTT_1257	553.0	642.0	N/D	14.9	N/D
Target	IC_{50} Value (nM)			Fold Change IC_{50} Value	
sFLT1-i13_2283	1.5	10.9	6.0	7.3	−1.8
sFLT1-e15a_2519	6.8	33.2	28.8	4.9	−1.2
HTT_462	91.6	142.3	N/D	1.6	N/D
HTT_10125	122.9	208.7	N/D	1.7	N/D
HTT_10146	64.3[a]	119.4	N/D	1.9	N/D
HTT_10150	44.4	166.8	88.4	3.8	−1.9
HTT_1219	164.0	213.0	N/D	1.3	N/D
HTT_1257	227.3	210.8	N/D	−1.1	N/D

Values are from Figures 2 and 3. When looking at differences, positive or negative values indicate an increase or decrease in values with the application of the indicated modification change. IC_{50} fold change was calculated by dividing the value obtained with the indicated modification change by the value obtained without it. However, if this number was <1, then the negative reciprocal is listed (e.g., 0.75, or a drop of 25% from the original value, is reported as −1.3-fold change). N/D, no data.
[a]The R^2 value for the fitted curve used to calculate the IC_{50} value <0.8.

and sFLT1-e15a_2519 siRNA guide and passenger strands and at positions 5 and 7 on the Tms of sFLT1-i13_2283 and sFLT1-e15a_2519 siRNA guide strand seed regions and their complementary RNAs.

Introduction of an Additional 2′-F in the Guide Strand Seed Does Not Measurably Impact Overall siRNA Duplex or Local Guide Strand Seed-Target Tms

In order to better define the mechanism(s) supporting our results, we first measured Tms for sFLT1-i13_2283 and sFLT1-e15a_2519 siRNA duplexes. We observed no measurable differences between siRNAs with 2′-F or 2′-OMe at position 20 or between siRNAs with 3′ end 2′-OMe and 2′-F or 2′-OMe at position 5 (Figure 4). Duplexes targeting *sFLT1-i13* with 14-mer passenger strands were included as a proof-of-principle control (Figure 4). As expected, Tms significantly decreased when a 14-mer instead of 15-mer passenger strand was used, but again, there was no change in Tm when 2′-OMe was included at the 3′ guide strand terminus or when 2′-F was added at guide strand position 5 (Figure 4). These results suggest that the negative impact of 2′-OMe at guide strand position 20 and the positive impact of 2′-F at guide strand position 5 are not due to changes in overall duplex Tm.

The increase of duplex Tm in the context of extensively chemically modified siRNAs could impede RISC loading by inhibiting unwinding of the passenger strand prior to its release.[14,15] However, if this were the primary mechanism influencing our results, then we would expect to see decreases in siRNA activity when 2′-F instead of 2′-OMe is included at guide strand positions 5 and 20, which is the opposite of what we observe. Therefore, these modification changes demonstrating no measurable impact on overall duplex Tm support our observations of their impacts on efficacy. Therefore, we next considered how including an additional 2′-F at guide strand position 5 might influence the Tm between an siRNA guide strand and its RNA target within the seed. 2′-F modification increases Tm, and this effect is more pronounced when consecutive nucleotides are modified.[3] Since initial seed interactions are impacted by local Tm, the stretch of 2′-F modifications from positions 4–6 could drive guide strand-loaded RISC to recognize its target with higher affinity and thus, positively impact target silencing. To test this, we synthesized single constructs that each included the following: (1) an 8-mer matching sFLT1-i13_2283 or sFLT1-e15a_2519 siRNA guide strand positions 1–8 in sequence and chemistry (i.e., cholesterol conjugated, with modifications as indicated in Figure 5A), (2) a UUCG tetraloop, and (3) a 7-mer unmodified RNA sequence reverse complementing sFLT1-i13_2283 or sFLT1-e15a_2519 positions 2–8 (i.e., the seed region) representing the RNA target. Initial experiments demonstrated insufficient hybridization between the 8- and 7-mer sequences to measure Tm, likely due to their short length and adenine A/ U richness. The additional stability offered by the UUCG tetraloop made it possible to measure Tms for these sequences within a hairpin structure. Measured Tms are therefore proxies for Tms for each guide strand and target within the seed. If the additions of 2′-F at guide strand position 5 or 7 resulted in changes in thermostability within the seed region, then relative changes in Tm would be observed using this strategy. However, the results of this experiment show negligible changes in Tm when these chemical modification patterns are applied. These data suggest that the changes in activity shown in Figures 3 and S2 are not likely due to changes in siRNA guide strand-target affinities in the seed.

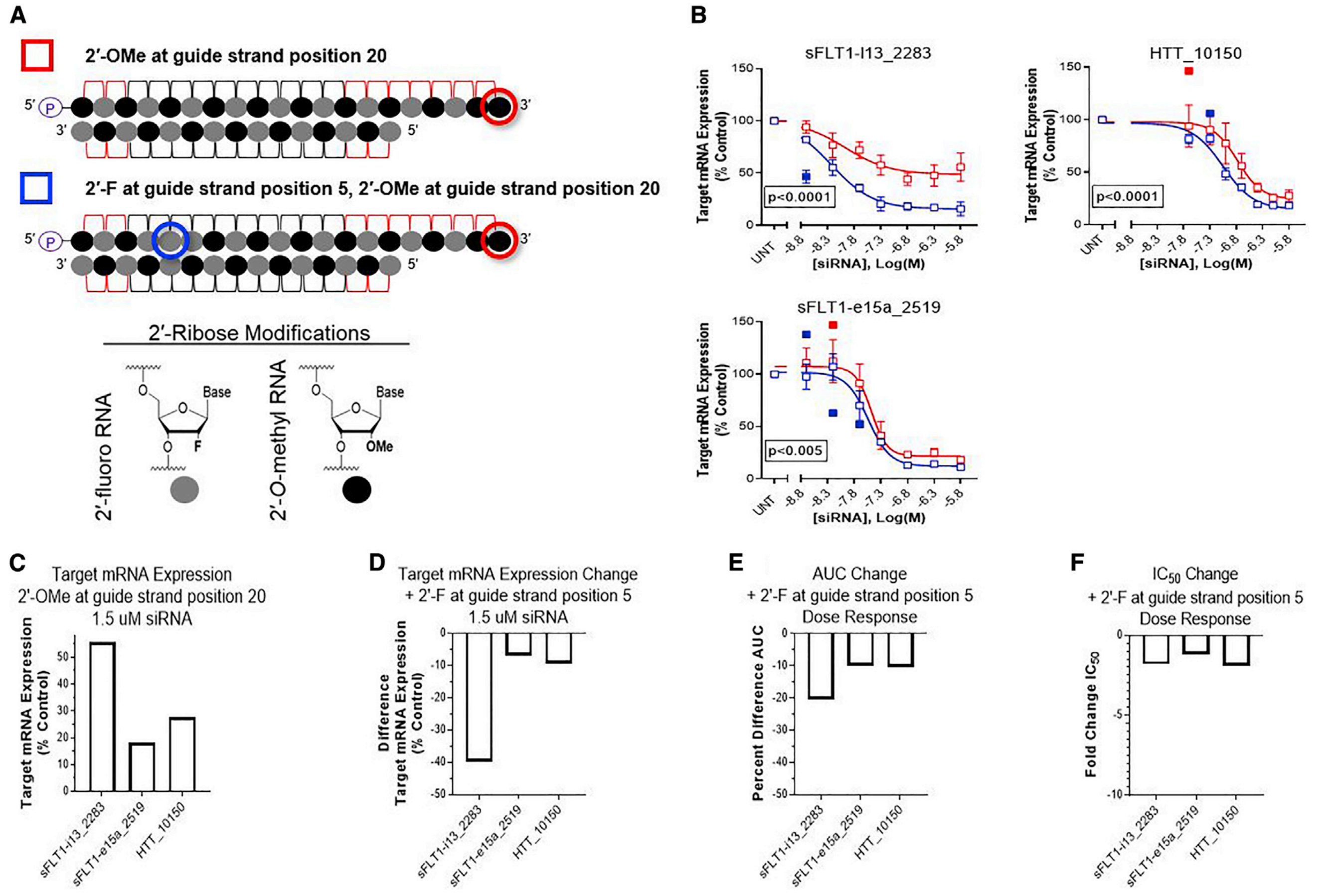

Figure 3. 2′-Fluoro at Position 5 of 20-mer Guide Strands Increases Activity of Fully Modified, Asymmetric siRNAs with 2′-*O*-Methyl at 3′ Guide Strand Termini

(A) Schematic representations of chemical modification scaffolds used; symbols next to each schematic are used in graphs shown in (B). Legend shows corresponding chemical structures for 2′-ribose modifications. (B) Dose-response results (n = 3, mean ± SD). Target name and start site of target sequence are indicated in each graph. HeLa cells were treated with siRNAs at concentrations shown for 72 h. mRNA levels were measured using the Dual-Glo Luciferase Assay System (*sFLT1-i13*, *sFLT1-e15a*) or Quantigene 2.0 RNA Assay (*HTT*) and calculated as a percent of those from untreated cell controls. Statistical outliers were excluded from analyses but are shown in the graphs as solid data points. p values displayed on each graph were calculated by two-way ANOVA. (C) Target mRNA expression with 1.5 μM of each siRNA with 2′-OMe at guide strand position 20. (D) Differences in target mRNA expression with 1.5 μM of each siRNA when 2′-F replaces 2′-OMe at guide strand position 5. (E) Percent differences in AUCs. (F) Fold changes in IC_{50} values, calculated by dividing the value obtained from siRNAs with 2′-F at guide strand position 5 by the value obtained from siRNAs with 2′-OMe at guide strand position 20; if this number was <1, then the negative reciprocal is shown. (D–F) Negative values indicate decreases in values when 2′-F replaces 2′-OMe at guide strand position 5.

Guide Strand Length Dictates the Extent to Which 3′ Terminal 2′-OMe Negatively Impacts the Activity of Fully Modified, Asymmetric siRNAs

There are multiple siRNA structures used in research and in the clinic, and the general belief is that guide strand length, within a range of 19–25 nt, minimally impacts siRNA efficacy. However, the type of 3′ terminal modification on a guide strand may affect its fitting within the PAZ domain of argonaute-2 (Ago2).[16,17] To investigate whether guide strand length impacts the observed negative effects of 3′ guide strand terminal 2′-OMe modification on siRNA activity, we synthesized 5 additional siRNA sequences as 19- and 21-mers with 2′-F or 2′-OMe 3′ terminal modification (Figures S3 and S4).

To our great surprise, when 2′-OMe was included at the 3′ end of 19- (Figure S3; Table S3) or 21-mer guide strands (Figure S4; Table S5), there were small or no significant negative impacts on siRNA efficacy. For siRNAs with 19-mer guide strands, HTT_10125 and HTT_1257 demonstrated statistically worse efficacy when 2′-OMe was included at the 3′ end of their guide strands instead of 2′-F (Figure S3). However, with a 19-mer guide strand, HTT_1257 showed less than a 6 percent difference in AUCs (Table S3), considerably less than the 15 percent difference demonstrated by this same sequence with a 20-mer guide strand (Table 1). For HTT_10125, the percent difference in AUCs indeed increases in the context of a 19 (Table S3)- versus 20 (Table 1)-mer guide strand, but the average difference in mRNA expression levels at the top 3 doses is 4 times lower (Table S4). For siRNAs with 21-mer guide strands,

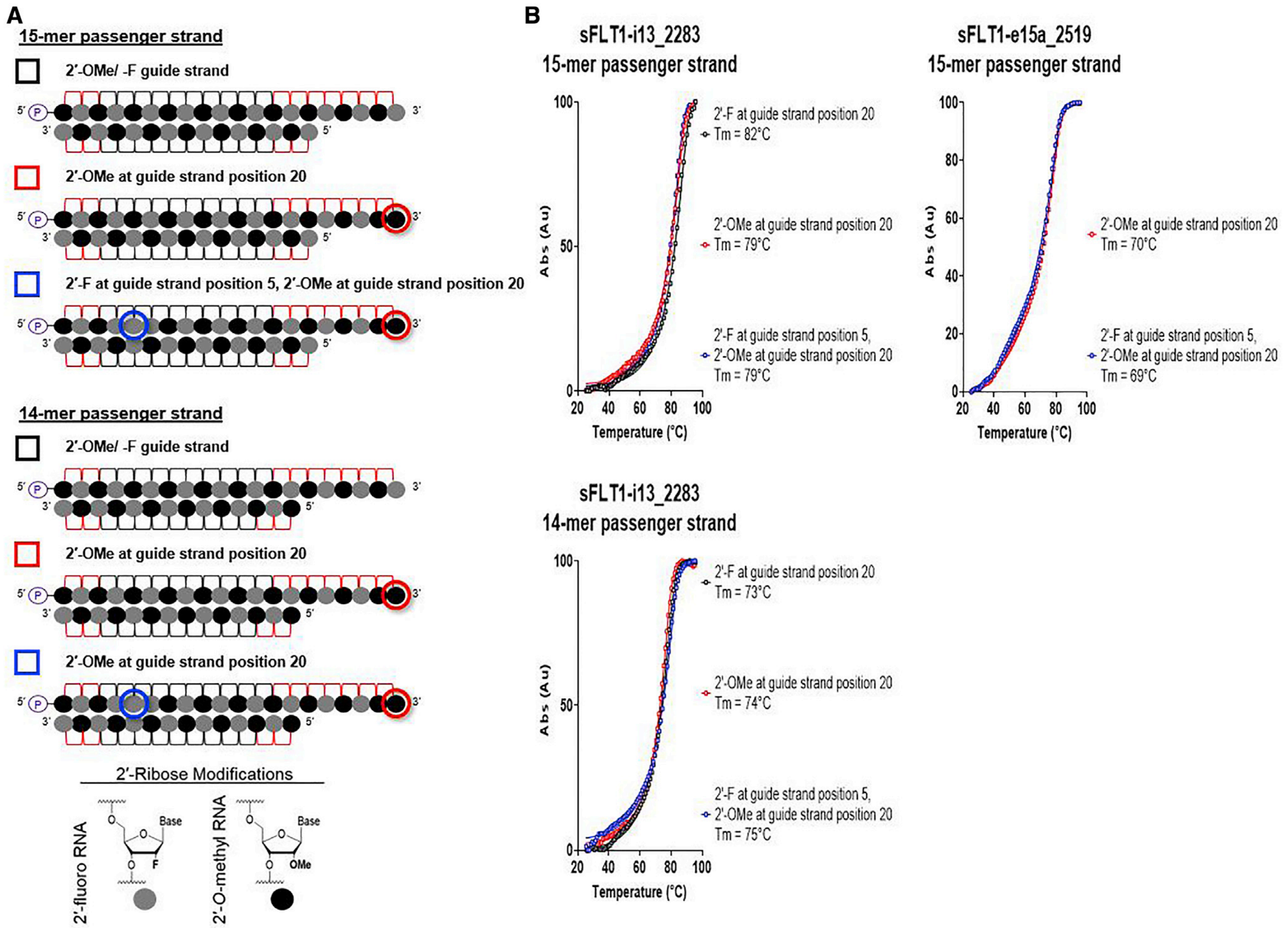

Figure 4. Introduction of 2′-*O*-Methyl at Guide Strand Position 20 and 2′-Fluoro at Guide Strand Position 5 Does Not Impact the Thermal Melting Temperatures of Fully Modified, Asymmetric siRNA Duplexes

(A) Schematic representations of guide strand chemical modification patterns used; symbols next to each schematic are used in graphs shown in (B). Legend shows corresponding chemical structures for 2′-ribose modifications. (B) Thermal melt curves for siRNAs. Target name, start site of target sequence, and length of paired passenger strands are indicated in graphs; Tm values are displayed in graph legends.

HTT_462 and HTT_1257 showed significant changes in efficacy when 2′-OMe, instead of 2′-F, was included at the 3′ end of their guide strands but performed better with guide strands containing 3′ terminal 2′-OMe instead of 2′-F, as evidenced by decreased mRNA expression at the top treatment dose and lower AUCs (Figure S4; Table S5). These results indicate that both siRNA structure and chemical modification pattern can cooperatively affect RISC interactions, and thus, both parameters should be considered when designing siRNAs.

We have a lot of experience using asymmetric siRNAs, which are efficiently internalized by cells when hydrophobically modified,[2] but the more symmetric 21-mer guide/19-mer passenger strand siRNA structure is widely used. The length of the guide strand determined the degree to which including 2′-OMe at 3′ guide strand termini decreased efficacy for asymmetric siRNAs—this modification negatively impacted activity for siRNAs with 20-mer guide and 15-mer passenger strands, but did not for 19-/ 15-mer or 21- /15-mer siRNAs. We next investigated if this modification affected the activity of more conventionally structured 21-mer guide/19-mer passenger siRNAs. Unfortunately, only one sequence maintained efficacy, whereas others silenced <50% of target mRNA (Figure S5; Table S6), making any small variations in activity between siRNAs with guide strands containing 3′ 2′-OMe or 2′-F difficult to detect. The structures of siRNAs variably impact different sequences,[2] and the sequences tested were originally selected for their activity in screens using the 20-mer guide/15-mer passenger strand asymmetric siRNA structure. Therefore, these sequences likely particularly benefitted from reduction of the passenger strand, which can contribute to more efficient passenger strand release and higher efficacy for some sequences.[2,14,15]

DISCUSSION

To date, there are no publications comparing the impacts of 3′ guide strand terminal 2′-OMe versus 2′-F modification on siRNA activity.

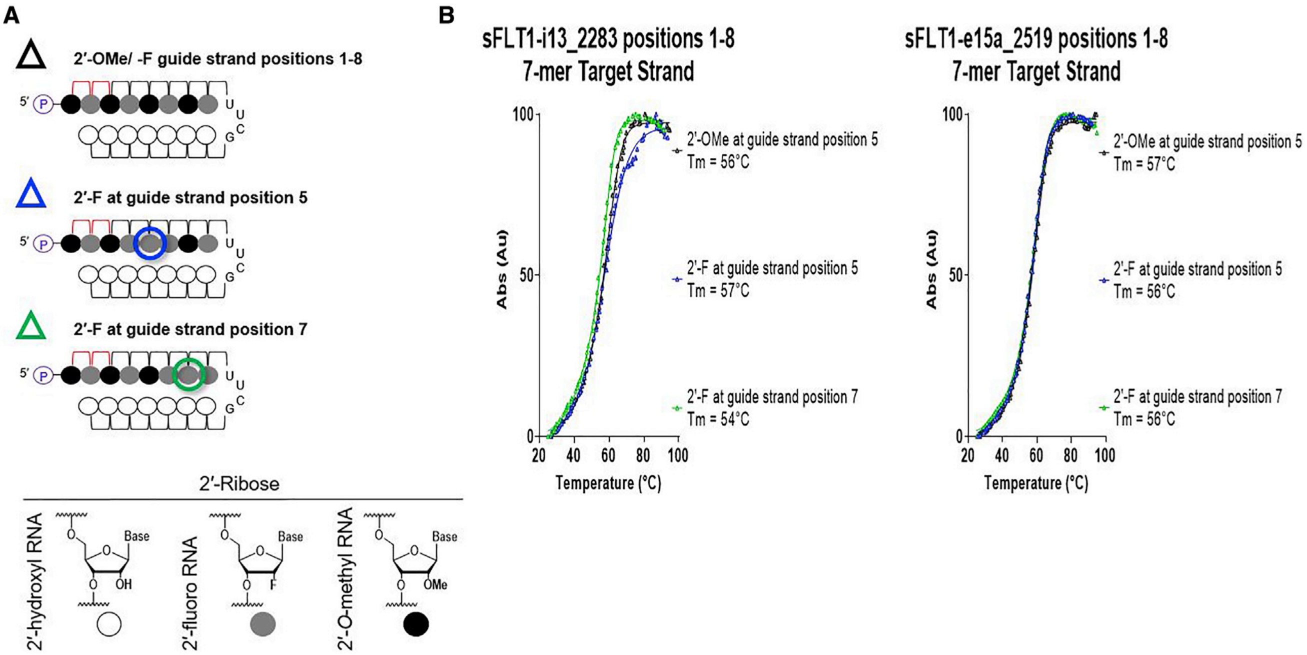

Figure 5. Introduction of an Additional 2′-Fluoro in the Guide Strand Seed Does Not Impact the Thermal Melting Temperatures of siRNA Guide Strands and Their RNA Targets within the Seed Region

(A) Schematic representations of guide strand chemical modification patterns used; symbols next to each schematic are used in graphs shown in (B). Legend shows corresponding chemical structures for 2′-ribose chemistries. (B) Thermal melt curves for siRNA guide strands (positions 1–8) and their targets within the seed region (i.e., guide strand positions 2–8). Target name and start site of target sequence are indicated in graphs; Tm values are displayed in graph legends.

Here, we demonstrate that including 2′-OMe instead of 2′-F at guide strand 3′ termini reduces siRNA efficacy and/or potency for multiple siRNA sequences and that this effect depends on the length of the guide strand. Interestingly, we also show that introducing an additional 2′-F modification in the guide strand seed partially compensates for this negative effect for 3 active sequences.

Our finding that the positive impact of 2′-F modification is specific to position 5 and not 7 is consistent with previous findings. Salomon et al.[18] report that nucleotides 2–5 more prominently influence the initial binding of guide strand-loaded RISC to its target RNA, whereas nucleotides 6–8 largely slow the subsequent dissociation of RISC from its target. Although these data support the inclusion of 2′-F at guide strand position 5, they may increase off-targeting activity—an important issue to consider, especially for high-affinity seed sequences.[19] The replacement of 2′-OMe with 2′-F at guide strand position 5 may also negatively impact duplex stability against nucleases, but this is difficult to determine experimentally, because all fully chemically modified siRNAs are very stable *in vitro* (with >24- to 48-h half-lives in 50% serum).[2,7,20] Long-term *in vivo* studies will be required to detect changes in nuclease stability caused by this single modification change within fully chemically modified siRNAs and will be necessary to fully assess the utility of this design feature.

The positive impact of 2′-F at guide strand position 5 may be due to it favorably positioning the guide strand in the Ago2-middle (MID) domain. We used PyMOL modeling to observe structural changes between siRNAs, with or without 2′-OMe, at this position. There is no crystal structure available for 20-mer siRNA guide strands, so we superimposed the crystal structure of human Ago2 loaded with a 21-mer modified siRNA onto the crystal structure of Ago2 with a 21-mer unmodified siRNA (Figure 6). The modified siRNA maintained the alternating 2′-OMe/-F pattern in the seed. In the context of this chemical pattern, position 5 is 2′-OMe. Figure 6B shows a slight shift in positioning of the modified guide strand, starting at position 5, relative to the unmodified guide strand within the Ago2-MID domain. Substitution with 2′-F, which is more similar in structure to the unmodified 2’-hydroxyl (2′-OH),[21] may better maintain the structure of native RISC complexed with naturally occurring, unmodified siRNA guide strands that are optimally configured within the seed region to recognize their mRNA target.

The negative impact of 2′-OMe at 3′ termini of 20-mer guide strands may be explained by its disruption to interactions between 3′ guide strand termini and the PAZ domain of Ago2. The 2′-OMe 3′ terminal modification is naturally occurring and likely enhances the stability of Piwi-interacting RNAs in *Drosophila melanogaster*.[22,23] We sought to visualize the structural impact of this modification in the context of human Ago2 and compare it to *Drosophila* Ago. With the use of PyMOL modeling, we superimposed the crystal structure of the *Drosophila* Ago-PAZ domain complexed with a 3′ end 2′-OMe 9-mer RNA onto the crystal structure of human Ago2 complexed

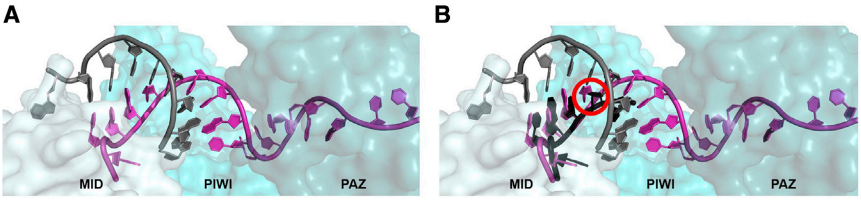

Figure 6. Replacing 2′-*O*-Methyl with 2′-Fluoro at Guide Strand Position 5 May Enhance siRNA-Target Interactions in the Seed

(A) Crystal structure of Ago2 loaded with a 21-mer unmodified siRNA guide strand (light magenta), optimally positioned to pair with its mRNA target (gray) in the seed (positions 2–8). (B) Image, as shown in (A), but also including a fully chemically modified 21-mer siRNA guide strand (black) with an alternating 2′-OMe/-F chemical pattern within its seed and 2′-OMe modification at position 5. Both siRNA sequences contain cysteine at guide strand position 5 (circled in red), but the chemically modified siRNA is positioned differently than the unmodified siRNA at this site, which may disrupt target binding.

with a 21-mer unmodified siRNA. Figure 7B shows *Drosophila* Ago with the 3′ end 2′-OMe 9-mer RNA and the unmodified siRNA guide strand. The closest contact to the 2′-OMe is 4.1 Å away. In human Ago2 (Figure 7C), the unmodified 2′-OH is also 4.1 Å apart from its closest contact. However, the 3′ end 2′-OMe 9-mer RNA more closely occupies the space of its closest contact, tyrosine 338, which is only 3.6 Å apart. This model suggests that the 3′ end 2′-OMe modified siRNA guide strand may not be properly accommodated by the Ago2-PAZ domain due to steric hindrance. Although this is only a model, it provides a potential explanation for the observed negative impact of the 3′ terminal 2′-OMe modification on human guide strand-loaded RISC function.

Naturally occurring siRNAs are 19–23 nt long,[24,25] but because there is no crystal structure available for siRNAs with 20-mer guide strands, we cannot visualize how the 3′-terminal nucleotide of a 20-mer guide strand interacts with Ago2-PAZ. Instead, Figure 7D displays Ago2 loaded with a 21-mer unmodified guide strand. The last two nucleotides, guide strand positions 20 and 21, are securely anchored into the PAZ domain-binding pocket. However, our 20-mer guide strand may be too short to similarly anchor itself in the PAZ domain once its 5′ end is anchored into the MID domain. The introduction of 2′-OMe at its terminal position (in place of 2′-F or 2′-OH) may exacerbate any already-existing issues it has anchoring into Ago2-PAZ. Since 2′-F is more structurally similar to 2′-OH, 20-mer guide strands with 3′-terminal 2′-F may be able to better contact residues important for anchoring within Ago2-PAZ.

The inclusion of 2′-OMe at the 3′ guide strand terminus may disrupt the proper positioning of the siRNA guide strand within human Ago2-PAZ, and in turn, this may suboptimally position the guide strand for finding its target and/or positioning its target for cleavage, which could explain our observation that including 2′-F at guide strand position 5 partially recovers activity for these guide strands. This hypothesis is consistent with the model proposed by Tomari and Zamore[26] and is supported by recent kinetics studies,[27] which show that an siRNA guide strand must first anchor within Ago2-PAZ to allow for initial target binding in the seed region but then must also detach from the PAZ domain to pair with its target past the seed region to position the target for cleavage. Consistent with this model, a 20-mer siRNA guide strand maintaining 2′-F at its 3′ end, may conform enough to the structure of native RNA to allow for its initial anchoring in the PAZ domain, and its shorter length may later facilitate its detachment from the PAZ domain. A 20-mer guide strand with 2′-OMe at its 3′ end may never successfully anchor into the PAZ domain, which may result in suboptimal positioning for interactions with its target.

The negative impact of the 3′ terminal 2′-OMe modification on RISC function may be specific to siRNAs with 20-mer guide strands. The pronounced negative effect on target silencing that we observed with this modification change was only demonstrated for siRNAs with 20-mer guide strands. The inclusion of 2′-OMe at the end of 19-mer guide strands did not negatively impact activity as much as it did for 20-mer guide strands. It is possible that 19-mer guide strands do not anchor into Ago2-PAZ as well as 20- or 21-mer guide strands, which may be why all but one sequence demonstrated overall reductions in efficacy when 19- instead of 20-mer guide strands were used (Figure S6; Table S7). If this is the case, then the contribution of the 3′ terminal modification on efficacy would be reduced for 19-mer guide strands, as was observed. In addition, siRNAs with 21-nt long guide strands were not negatively affected by 3′ terminal 2′-OMe; for two sequences, this modification statistically improved efficacy ($p < 0.0001$).

Since lead clinical candidates possess 2′-OMe modifications at the 3′ guide strand termini, the observation that 2′-OMe modification at 3′ guide strand termini negatively affects RISC function may be somewhat surprising. However, most clinical compounds possess longer (i.e., 21- to 23-mer) guide strands. Indeed, Alnylam's "standard," "enhanced," and "advanced enhanced stabilization" chemistries utilize 23-mer guide strands, which likely anchor in the PAZ domain more efficiently than 20-mer guide strands. If this is the case, then it may be beneficial for clinical candidates with longer guide strands to contain 2′-OMe at the 3′ end to facilitate detachment.

Taken together, our results indicate a cooperative impact of siRNA structure, chemical modification pattern, and sequence on RISC interactions and highlight the importance of considering all 3 parameters when designing siRNAs for screening purposes. They also offer improved design rules for 20-/15-mer siRNAs maintaining the alternating 2′-OMe/-F guide strand pattern described by Allerson et al.[7] This chemical pattern is widely used in research, because it affords activity to many siRNA sequences, the asymmetric structure evaluated in these studies has been previously demonstrated to be efficiently internalized into cells,[2] and we show that a 20-mer guide strand

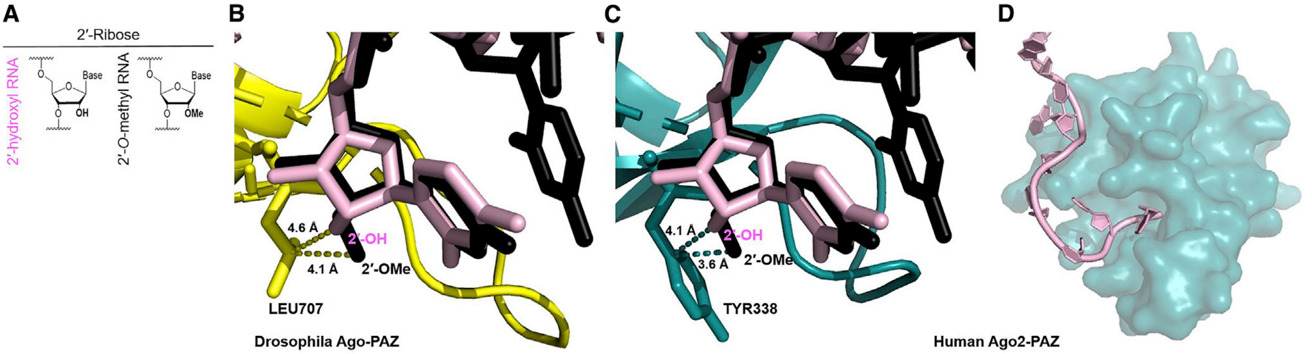

Figure 7. Including 2′-*O*-Methyl at the 3′ End of an siRNA Guide Strand May Disrupt Its Anchoring in Ago2-PAZ

(A) Legend shows corresponding chemical structures for 3′ end 2′-ribose chemistries. (B) Crystal structure of the *Drosophila* Ago-PAZ domain (yellow), loaded with a 9-mer small RNA (black) with 2′-OMe at its 3′ end. Image also includes a 21-mer unmodified siRNA guide strand (raspberry). The distances between the 3′ ends of each small RNA and the closest residue in *Drosophila* Ago (LEU707) are shown. (C) Crystal structure of human Ago2-PAZ (deep teal), loaded with a 21-mer unmodified siRNA guide strand (light pink). Image also includes a 9-mer small RNA (black) with 2′-OMe at its 3′ end. The distances between the 3′ ends of each small RNA and the closest residue in Ago2 (TYR338) are shown. (D) Crystal structure of human Ago2-PAZ (deep teal), loaded with a 21-mer unmodified siRNA guide strand (light pink) showing positions 20 and 21 anchored into the binding pocket of the PAZ domain.

provides similar or better efficacy than its comparable 21-mer guide strand for multiple siRNA sequences (Figure S7; Table S8). In the contexts of this chemical-modification pattern, siRNA structure, and guide strand length, the incorporation of 2′-F at guide strand positions 5 and 20 may be a better design rule than the standard 2′-OMe/-F alternating pattern for fully modified siRNAs used for research applications.

The application of chemical modifications to siRNAs impacts their interactions with other proteins, besides Ago2, that were not evaluated here. For example, chemical modifications inhibit siRNA recognition by the innate immune system and degradation by nucleases. Both 2′-F and 2′-OMe modifications have been previously demonstrated to abrogate activation of Toll-like receptors;[28] therefore, we do not expect these types of interactions to contribute to our observed effects. However, these modifications may differentially impact nuclease stability. Thus, in the future, it will be important to elucidate the impacts of 2′-OMe, 2′-F, and 2′-OH at 3′ guide strand termini on 3′ stabilization *in vivo*. It will also be interesting to look at the impacts of other chemical modifications widely used in research for 3′ stabilization, like inverted deoxythymidine, in the context of our 20-mer guide strands, on both siRNA efficacy and guide strand 3′ stabilization. The primary nuclease activity against single-stranded 3′ siRNA guide strand overhangs in serum is processive 3′-exonuclease digestion,[29,30] but the primary RNA degradation mechanism by most RNases occurs through the attack of ribose 2′-OH on its 3′ phosphate backbone, and the 3′ phosphate is not naturally present at the 3′ terminus. Therefore, the stabilizing effects of 2′-F versus 2′-OMe at this position may be relatively similar.

MATERIALS AND METHODS

Oligonucleotide Synthesis, Deprotection, and Purification

Oligonucleotides were synthesized using standard and modified (2′-F, 2′-OMe) phosphoramidite, solid-phase synthesis conditions using a MerMade 12 (BioAutomation, Irving, TX, USA) and Dr. Oligo 48 Medium Throughput Oligo Synthesizer (Biolytic; #403-104814). Cholesterol-conjugated oligonucleotides were synthesized on modified solid support (Chemgenes; #N-9166-05). Oligonucleotides were removed from controlled pore glass (CPG), deprotected, and purified by high-performance liquid chromatography (HPLC), as described previously,[31] or ethanol precipitated (for sequences, chemical modification patterns, and purification methods, see Table S10). Purified oligonucleotides were passed over a Hi-Trap cation exchange column to exchange the counter-ion with sodium. All oligonucleotide identites were confirmed by HPLC-mass spectrometry.

Cell Culture (HeLa and WM-115 cells)

HeLa cells (ATCC; #CCL-2) were maintained in Dulbecco's modified Eagle's medium (DMEM) (Cellgro; #10-013CV), and WM-115 cells (ATCC; CRL-1676) were maintained in Eagle's minimum essential medium (EMEM) (Sigma-Aldrich; M0643). The media were supplemented with 9% fetal bovine serum (FBS) (Gibco; #26140), and all cells were grown at 37°C and 5% CO_2. Cells were split every 2 to 7 days and discarded after fifteen passages.

Direct Delivery (i.e., Passive Uptake) of Oligonucleotides

sFLT1-i13_2283 and sFLT1-e15a_2519, HeLa Cells

cDNA sequences, corresponding to 20-nt-long, unique regions of sFLT1-i13 and sFLT1-e15a target mRNAs, were cloned into a psiCheck-2 vector (Promega, Madison, WI, USA; C8021), according to the manufacturer's protocol. HeLa cells were plated on a 10-cm dish and transfected with 24 μg of the psiCheck-2 plasmid using Lipofectamine 2000 (Invitrogen, Carlsbad, CA, USA; 11668019), according to the manufacturer's protocol. Cells were transferred to 96-well cell-culture plates, 24 h later.

All Targets, All Cells

HeLa cells were diluted in DMEM containing 6% FBS, and WM-115 cells were diluted in EMEM containing 4.5% FBS to 8,000–10,000

(HeLa) or 25,000 (WM-115) cells per 50 μL. siRNAs were diluted to twice the final maximum test concentration in OptiMEM (Carlsbad, CA, USA; 31985-088) and serially diluted to create 7-point dose responses. 50 μL of diluted siRNA was added to 50 μL of cells, resulting in a final concentration of 1.5 μM siRNA for the maximum dose and 3% or 2.25% FBS. Cells were incubated for 72 h at 37°C and 5% CO_2.

Method for Quantitative Analysis of Target mRNA Expression

sFLT1-i13_2283 and sFLT1-e15a_2519, HeLa Cells

mRNA was quantified using the Dual-Glo Luciferase Assay System, according to the manufacturer's protocol (Promega; #E2940). Luminescence was detected on a Veritas Luminometer (Promega; #998-9100) or a Tecan M1000 (Tecan, Morrisville, NC, USA). For each cell-treatment plate, data were normalized to control reporter firefly luciferase (fLuc) and plotted as a percentage of the mean results from untreated cells.

sFLT1-i13_2283 and sFLT1-e15a_2519, WM-115 cells; HTT_X, HeLa cells

mRNA was quantified using the QuantiGene 2.0 assay kit (Affymetrix; QS0011). Cells were lysed in 250 μL diluted lysis mixture composed of 1 part lysis mixture (Affymetrix; 13228), 2 parts H_2O, and 0.167 μg/μL proteinase K (Affymetrix; QS0103) for 30 min at 55°C. Probe sets for human *sFLT1-i13*, *sFLT1-e15a*, *HTT*, and *HPRT* (Affymetrix; SA-50459, SA-50496, SA-50339, SA-10030) were diluted and used according to the manufacturer's recommended protocol. Cell lysates were mixed thoroughly before 20–60 μL of lysate and 20 μL of probe set mixture were added to each well of a capture plate in triplicate. 20–60 μL of lysis mixture, diluted in 2 parts H_2O, was also added, such that each well contained 100 μL total. For each cell-treatment plate, *HPRT* was used as a normalization and/ or visual control, and data were plotted as a percentage of the mean results from untreated cells.

Statistical Analyses

Dose-response data were analyzed using GraphPad Prism 8.3.0 software (GraphPad Software, San Diego, CA, USA). Concentration-dependent IC_{50} curves were fitted using the log(inhibitor) versus response – variable slope (four parameters) method. The lower limit of the curve was set to >0, and the upper limit of the curve was set to >95. Statistical outliers were identified using Q = 1% and excluded from calculation of the fitted curve but shown on the graphs as solid points. If the R-squared value for the fitted curve was <0.8, then the IC_{50} value is marked in its table, and its curve is shown as a dashed line in its graph. If the Prism calculated IC_{50} value was >1.5 μM (i.e., greater than the top treatment dose), then the IC_{50} value was not included in its table, and IC_{50} fold change was not calculated. p values were calculated using two-way ANOVA (analysis excluded statistical outliers identified as previously described). AUCs were calculated using automatic software settings, i.e., baseline: y = 0, minimum peak height: ignore peaks < 10% of the distance from Y_{min} to Y_{max} (analysis excluded statistical outliers identified, as previously described).

Tm Experiments and Calculations

Samples were diluted in 1× Dulbecco's phosphate-buffered saline (PBS) (Sigma-Aldrich; D8662), and A_{260} values were collected at 1°C/1 min intervals over three ramps: 25°C–95°C, 95°C–25°C, and 25°C–95°C with 1× PBS blank correction using Agilent Technologies (Agilent) Cary 100 Series UV-Vis Spectrophotometer (Agilent; #G9821A). The readings from the third ramp for data shown in Figure 4 or the second ramp for data shown in Figure 5 were minimum-maxiumum (min-max) normalized and plotted. Tm was calculated using GraphPad Prism 8.3.0 software.

PyMOL Modeling

For all figures, the crystal structures of human Ago2 loaded with a 21-mer unmodified siRNA guide strand (PDB: 4W5N), human Ago2 loaded with a 21-mer unmodified siRNA guide strand bound to its target (PDB: 4W5Q), and human Ago2 loaded with a 21-mer modified siRNA guide strand (PDB: 5JS2) were loaded into PyMOL (Schrödinger)

Ago2 (chain A) from PDB:4W5Q and PDB: 5JS2 were superimposed onto Ago2 (chain A) from PDB: 4W5N and then hidden, leaving only Ago2 from PDB: 4W5N (the most thoroughly resolved Ago2 structure) and guide strands from PDB: 4W5N, PDB: 4W5Q, and PDB: 5JS2 and the target from PDB: 4W5Q. The MID (Gly445-Ile577), PIWI (Leu579-Ala859), and PAZ (Pro229-Val347) domains of human Ago2 from PDB: 4W5N were selected and colored pale cyan, cyan, and deep teal, respectively, and all but these three domains in Ago2 from PDB: 4W5N (chain A) were hidden.

The 21-mer unmodified guide strand from PDB: 4W5N (nucleotides 1–7 and 12–21 resolved) was light pink. The 21-mer unmodified guide strand from PDB: 4W5Q (nucleotides 1–18 resolved) was light magenta. The guide strand-bound target from PDB: 4W5Q (nucleotides 1–9 resolved) was gray 50. The 21-mer modified guide strand from PDB: 5JS2 (residues 1–6 resolved) was black.

The crystal structure of *Drosophila* Ago-PAZ loaded with a 9-mer 3′ end 2′-O-methylated small RNA (PDB: 3MJ0) was loaded into PyMOL. *Drosophila* Ago-PAZ (chain A) from PDB: 3MJ0 was superimposed onto the Ago2-PAZ domain in 4W5N and colored yellow. The 9-mer 3′ end 2′-O-methylated small RNA (nucleotides 1–9 resolved) was black.

For the Graphical Abstract, the MID, PIWI, and PAZ domains of Ago2, nucleotides 19–20 in the 21-mer unmodified guide strand from PDB: 4W5N, and all resolved nucleotides in the 21-mer unmodified guide strand and bound target from PDB: 4W5Q are shown.

For Figure 6A, the MID, PIWI, and PAZ domains of Ago2 from PDB: 4W5N and all resolved nucleotides in the 21-mer unmodified guide strand and bound target from PDB: 4W5Q are shown. For Figure 6B, the MID, PIWI, and PAZ domains of Ago2 from PDB: 4W5N, all resolved nucleotides in the 21-mer unmodified guide strand and

bound target from PDB: 4W5Q and all resolved nucleotides in the 21-mer modified guide strand from PDB: 5JS2 are shown.

For Figure 7B, Ago-PAZ and all resolved nucleotides in the 9-mer 3′ end 2′-O-methylated small RNA from PDB: 3MJ0 and all resolved nucleotides in the 21-mer unmodified guide strand from PDB: 4W5N are shown. For Figure 7C, the PAZ domain of Ago2, all resolved nucleotides in the 21-mer unmodified guide strand from PDB: 4W5N, and all resolved nucleotides in the 9-mer 3′ end 2′-O-methylated small RNA from PDB: 3MJ0 are shown. For Figure 7D, the PAZ domain of Ago2 and all resolved nucleotides in the 21-mer unmodified guide strand from PDB: 4W5N are shown.

SUPPLEMENTAL INFORMATION

Supplemental Information can be found online at https://doi.org/10.1016/j.omtn.2020.05.010.

AUTHOR CONTRIBUTIONS

S.M.D. designed and conducted *in vitro* concentration response experiments and drafted the manuscript. J.S. synthesized all oligonucleotides for 19-/15-mer, 21-/15-mer, and 21-/19-mer *HTT* targeting siRNAs (Figures S3–S7); all 20-/15-mer *HTT* siRNAs in Figure 2, except HTT_10150; and oligonucleotides for thermal melt experiments, shown in Figure 5. L.V. contributed to preliminary experiments (not shown) by synthesizing 8-mer siRNA guide strands and 7-mer RNA target strands (later synthesized by J.S. joined by tetraloop; see Figure 5), hybridizing them, purifying them by hydrophilic interaction liquid chromatography and nondenaturing ion pairing-reverse-phase chromatography, and investigating their hybridization efficiencies via mass spectrometry analysis. M.R.H. and D.E. synthesized oligonucleotides for sFLT1-i13, sFLT1-e15a, and 20-/15-mer HTT_10150 siRNAs (Figures 2, 3, 4, S1, S2, and S7). E.K. performed *in vitro* concentration response experiments for HTT_10150 20-/15-mer siRNA (Figures 2 and S7). A.A.T. and J.F.A. conceived the chemical modification patterns used for 20-/15-mer sFLT1-i13, sFLT1-e15a, and HTT_10150 siRNAs (Figures 2, 3, S1, S2, and S7). A.K. guided the study designs and helped to draft the manuscript.

CONFLICTS OF INTEREST

The authors declare no competing interests.

ACKNOWLEDGMENTS

All work was completed at the University of Massachusetts Medical School, Worcester, MA 01605. This study was supported by grants 1R35GM131839-01; 5R01HD086111-02; S10 OD 020012-01; CHDI Foundation (RecID A-5038).

REFERENCES

1. Fire, A., Xu, S., Montgomery, M.K., Kostas, S.A., Driver, S.E., and Mello, C.C. (1998). Potent and specific genetic interference by double-stranded RNA in Caenorhabditis elegans. Nature *391*, 806–811.
2. Hassler, M.R., Turanov, A.A., Alterman, J.F., Haraszti, R.A., Coles, A.H., Osborn, M.F., Echeverria, D., Nikan, M., Salomon, W.E., Roux, L., et al. (2018). Comparison of partially and fully chemically-modified siRNA in conjugate-mediated delivery in vivo. Nucleic Acids Res. *46*, 2185–2196.
3. Cummins, L.L., Owens, S.R., Risen, L.M., Lesnik, E.A., Freier, S.M., McGee, D., Guinosso, C.J., and Cook, P.D. (1995). Characterization of fully 2′-modified oligoribonucleotide hetero- and homoduplex hybridization and nuclease sensitivity. Nucleic Acids Res. *23*, 2019–2024.
4. Foster, D.J., Brown, C.R., Shaikh, S., Trapp, C., Schlegel, M.K., Qian, K., Sehgal, A., Rajeev, K.G., Jadhav, V., Manoharan, M., et al. (2018). Advanced siRNA Designs Further Improve In Vivo Performance of GalNAc-siRNA Conjugates. Mol. Ther. *26*, 708–717.
5. Shmushkovich, T., Monopoli, K.R., Homsy, D., Leyfer, D., Betancur-Boissel, M., Khvorova, A., and Wolfson, A.D. (2018). Functional features defining the efficacy of cholesterol-conjugated, self-deliverable, chemically modified siRNAs. Nucleic Acids Res. *46*, 10905–10916.
6. Chiu, Y.L., and Rana, T.M. (2003). siRNA function in RNAi: a chemical modification analysis. RNA *9*, 1034–1048.
7. Allerson, C.R., Sioufi, N., Jarres, R., Prakash, T.P., Naik, N., Berdeja, A., Wanders, L., Griffey, R.H., Swayze, E.E., and Bhat, B. (2005). Fully 2′-modified oligonucleotide duplexes with improved in vitro potency and stability compared to unmodified small interfering RNA. J. Med. Chem. *48*, 901–904.
8. Geary, R.S., Yu, R.Z., and Levin, A.A. (2001). Pharmacokinetics of phosphorothioate antisense oligodeoxynucleotides. Curr. Opin. Investig. Drugs *2*, 562–573.
9. Watanabe, T.A., Geary, R.S., and Levin, A.A. (2006). Plasma protein binding of an antisense oligonucleotide targeting human ICAM-1 (ISIS 2302). Oligonucleotides *16*, 169–180.
10. Ly, S., Navaroli, D.M., Didiot, M.C., Cardia, J., Pandarinathan, L., Alterman, J.F., Fogarty, K., Standley, C., Lifshitz, L.M., Bellve, K.D., et al. (2017). Visualization of self-delivering hydrophobically modified siRNA cellular internalization. Nucleic Acids Res. *45*, 15–25.
11. Manoharan, M., Akinc, A., Pandey, R.K., Qin, J., Hadwiger, P., John, M., Mills, K., Charisse, K., Maier, M.A., Nechev, L., et al. (2011). Unique gene-silencing and structural properties of 2′-fluoro-modified siRNAs. Angew. Chem. Int. Ed. Engl. *50*, 2284–2288.
12. Deleavey, G.F., Watts, J.K., and Damha, M.J. (2009). Chemical modification of siRNA. Curr. Protoc. Nucleic Acid Chem. *Chapter 16*, Unit 16.3.
13. Kawasaki, A.M., Casper, M.D., Freier, S.M., Lesnik, E.A., Zounes, M.C., Cummins, L.L., Gonzalez, C., and Cook, P.D. (1993). Uniformly modified 2′-deoxy-2′-fluoro phosphorothioate oligonucleotides as nuclease-resistant antisense compounds with high affinity and specificity for RNA targets. J. Med. Chem. *36*, 831–841.
14. Rand, T.A., Petersen, S., Du, F., and Wang, X. (2005). Argonaute2 cleaves the anti-guide strand of siRNA during RISC activation. Cell *123*, 621–629.
15. Matranga, C., Tomari, Y., Shin, C., Bartel, D.P., and Zamore, P.D. (2005). Passenger-strand cleavage facilitates assembly of siRNA into Ago2-containing RNAi enzyme complexes. Cell *123*, 607–620.
16. Valenzuela, R.A., Onizuka, K., Ball-Jones, A.A., Hu, T., Suter, S.R., and Beal, P.A. (2016). Guide Strand 3′-End Modifications Regulate siRNA Specificity. ChemBioChem *17*, 2340–2345.
17. Hong, S.W., Park, J.H., Yun, S., Lee, C.H., Shin, C., and Lee, D.K. (2014). Effect of the guide strand 3′-end structure on the gene-silencing potency of asymmetric siRNA. Biochem. J. *461*, 427–434.
18. Salomon, W.E., Jolly, S.M., Moore, M.J., Zamore, P.D., and Serebrov, V. (2015). Single-Molecule Imaging Reveals that Argonaute Reshapes the Binding Properties of Its Nucleic Acid Guides. Cell *162*, 84–95.
19. Janas, M.M., Schlegel, M.K., Harbison, C.E., Yilmaz, V.O., Jiang, Y., Parmar, R., Zlatev, I., Castoreno, A., Xu, H., Shulga-Morskaya, S., et al. (2018). Selection of GalNAc-conjugated siRNAs with limited off-target-driven rat hepatotoxicity. Nat. Commun. *9*, 723.
20. Choung, S., Kim, Y.J., Kim, S., Park, H.-O., and Choi, Y.-C. (2006). Chemical modification of siRNAs to improve serum stability without loss of efficacy. Biochem. Biophys. Res. Commun *342*, 919–927.
21. Khvorova, A., and Watts, J.K. (2017). The chemical evolution of oligonucleotide therapies of clinical utility. Nat. Biotechnol. *35*, 238–248.

22. Yu, B., Yang, Z., Li, J., Minakhina, S., Yang, M., Padgett, R.W., Steward, R., and Chen, X. (2005). Methylation as a crucial step in plant microRNA biogenesis. Science *307*, 932–935.
23. Ghildiyal, M., and Zamore, P.D. (2009). Small silencing RNAs: an expanding universe. Nat. Rev. Genet. *10*, 94–108.
24. Zamore, P.D., Tuschl, T., Sharp, P.A., and Bartel, D.P. (2000). RNAi: double-stranded RNA directs the ATP-dependent cleavage of mRNA at 21 to 23 nucleotide intervals. Cell *101*, 25–33.
25. Elbashir, S.M., Lendeckel, W., and Tuschl, T. (2001). RNA interference is mediated by 21- and 22-nucleotide RNAs. Genes Dev *15*, 188–200.
26. Tomari, Y., and Zamore, P.D. (2005). Perspective: machines for RNAi. Genes Dev. *19*, 517–529.
27. Deerberg, A., Willkomm, S., and Restle, T. (2013). Minimal mechanistic model of siRNA-dependent target RNA slicing by recombinant human Argonaute 2 protein. Proc. Natl. Acad. Sci. USA *110*, 17850–17855.
28. Sioud, M. (2006). Single-stranded small interfering RNA are more immunostimulatory than their double-stranded counterparts: a central role for 2′-hydroxyl uridines in immune responses. Eur. J. Immunol. *36*, 1222–1230.
29. Eder, P.S., DeVine, R.J., Dagle, J.M., and Walder, J.A. (1991). Substrate specificity and kinetics of degradation of antisense oligonucleotides by a 3′ exonuclease in plasma. Antisense Res. Dev. *1*, 141–151.
30. Zou, Y., Tiller, P., Chen, I.-W., Beverly, M., and Hochman, J. (2008). Metabolite identification of small interfering RNA duplex by high-resolution accurate mass spectrometry. Rapid Commun. Mass Spectrom. *22*, 1871–1881.
31. Alterman, J.F., Hall, L.M., Coles, A.H., Hassler, M.R., Didiot, M.-C., Chase, K., Abraham, J., Sottosanti, E., Johnson, E., Sapp, E., et al. (2015). Hydrophobically modified siRNAs silence huntingtin mRNA in primary neurons and mouse brain. Mol. Ther. Nucleic Acids *4*, e266.

OMTN, Volume *21*

Supplemental Information

2′-*O*-Methyl at 20-mer Guide Strand 3′ Termini May Negatively Affect Target Silencing Activity of Fully Chemically Modified siRNA

Sarah M. Davis, Jacquelyn Sousa, Lorenc Vangjeli, Matthew R. Hassler, Dimas Echeverria, Emily Knox, Anton A. Turanov, Julia F. Alterman, and Anastasia Khvorova

Supplemental Figures

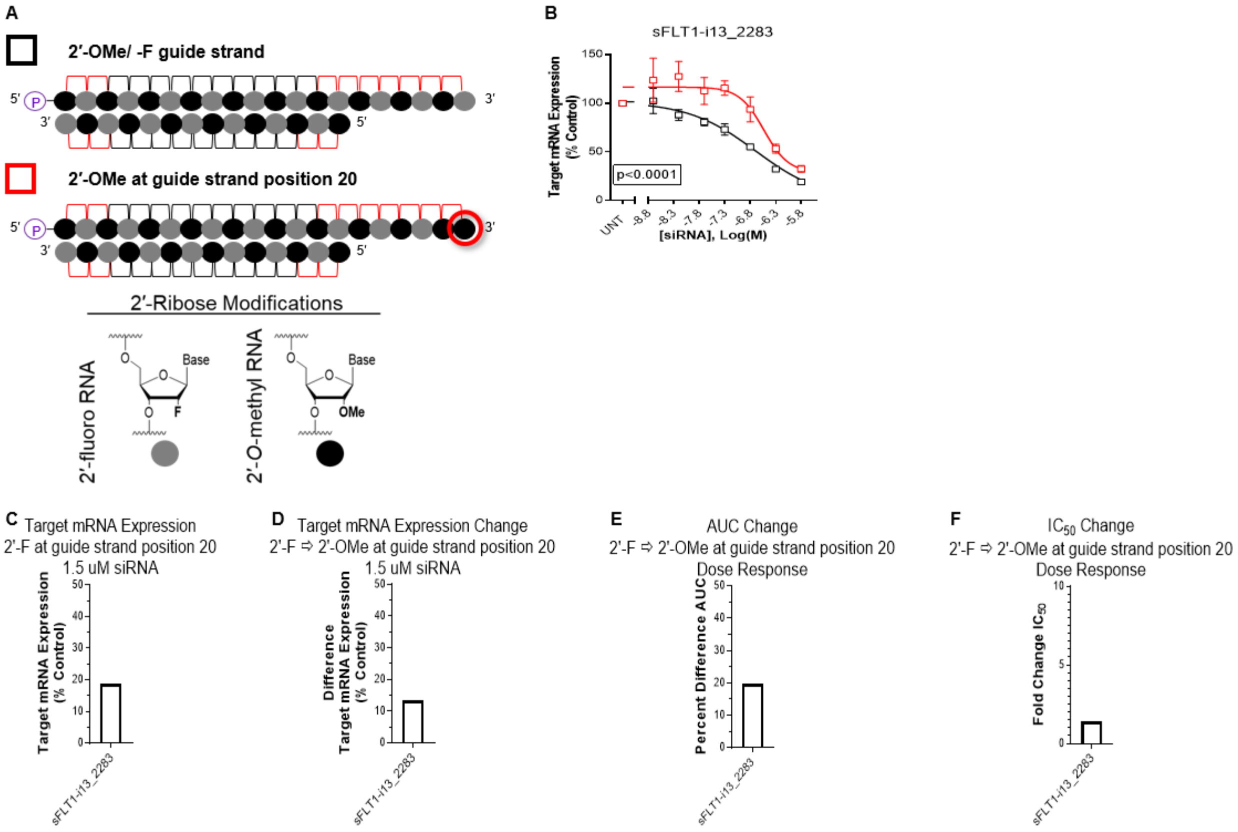

Figure S1. 2′-*O*-methyl at 3′ termini of 20-mer guide strands decreases activity of fully modified, asymmetric siRNAs with 14-mer passenger strands. (A) Schematic representations of chemical modification scaffolds used; symbols next to each schematic are used in graphs shown in (B). Legend shows corresponding chemical structures for 2′-ribose modifications. (B) Dose response results (n=3, mean ± SD). Target name and start site of target sequence indicated in each graph. WM-115 cells were treated with siRNAs at concentrations shown for 72 hours. mRNA levels were measured using the Quantigene 2.0 RNA Assay and calculated as a percent of those from untreated cell controls. p-values displayed on each graph calculated by two-way ANOVA. (C) Target mRNA expression with 1.5 µM of each siRNA with 2′-F at guide strand position 20. (D) Differences in target mRNA expression with 1.5 µM of each siRNA when 2′-OMe replaces 2′-F at guide strand position 20. (E) Percent differences in AUCs. (F) Fold changes in IC_{50} values, calculated by dividing the value obtained from siRNAs with 2′-OMe at guide strand position 20 by the value obtained from siRNAs with 2′-F at guide strand position 20. (D, E, F) Positive values indicate increases in values when 2′-OMe replaces 2′-F at guide strand position 20.

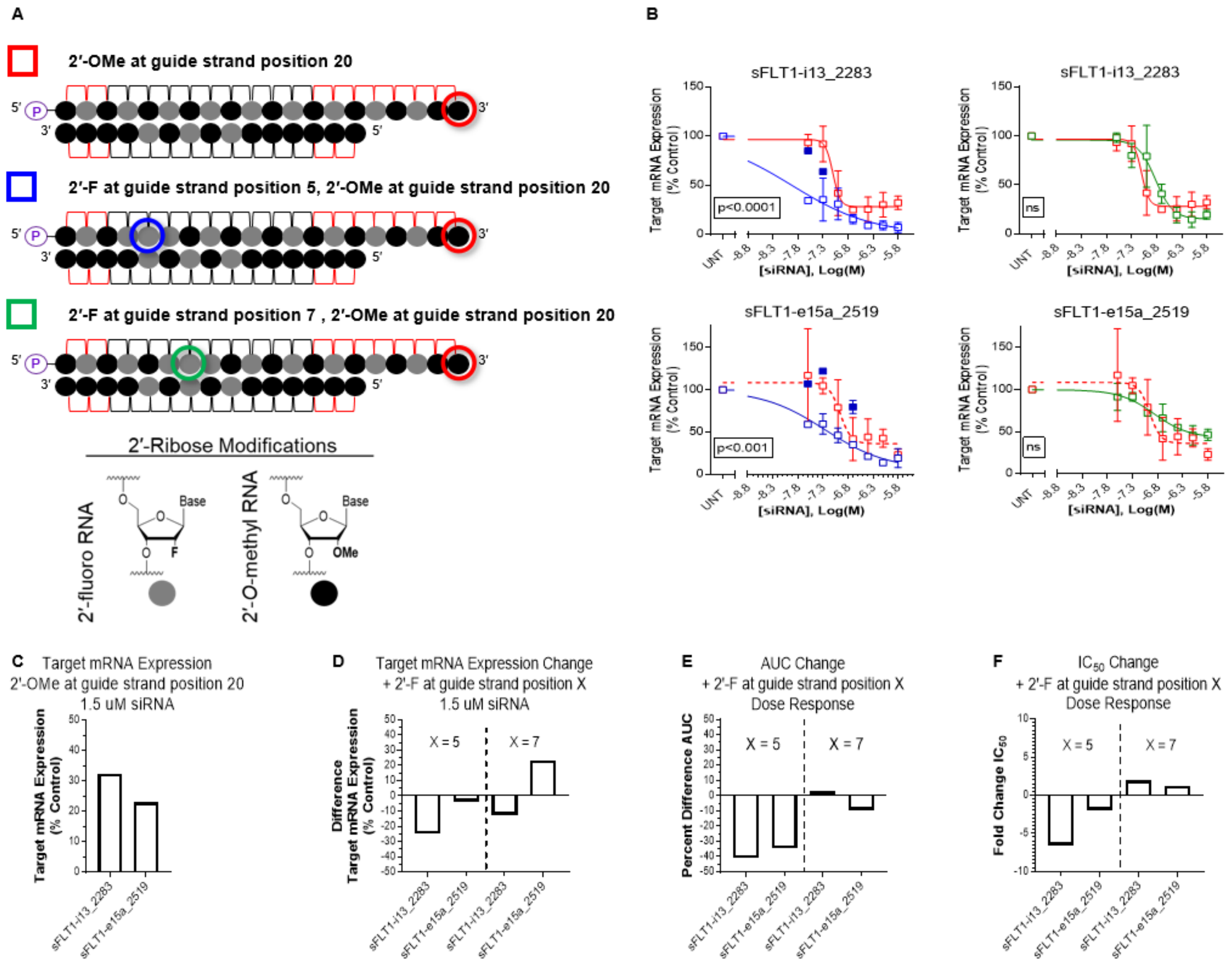

Figure S2. 2′-fluoro at position 5 but not 7 of 20-mer guide strands increases activity of fully modified, asymmetric siRNAs with 2′-*O*-methyl at 3′ guide strand termini. (A) Schematic representations of chemical modification scaffolds used; symbols next to each schematic are used in graphs shown in (B). Legend shows corresponding chemical structures for 2′-ribose modifications. (B) Dose response results (n=3, mean ± SD). Target name and start site of target sequence indicated in each graph. HeLa cells were treated with siRNAs at concentrations shown for 72 hours. mRNA levels were measured using the Dual-Glo® Luciferase Assay System and calculated as a percent of those from untreated cell controls. Statistical outliers are excluded from analysis, but are shown in the graphs as solid data points. Non-linear regression curves with $R^2<0.8$ are displayed as dashed rather than solid lines. p-values displayed on each graph calculated by two-way ANOVA. (C) Target mRNA expression with 1.5 μM of each siRNA with 2′-OMe at guide strand position 20. (D) Differences in target mRNA expression with 1.5 μM of each siRNA when 2′-F replaces 2′-OMe at guide strand position 5 or 7. (E) Percent differences in AUCs. (F) Fold changes in IC_{50} values, calculated by dividing the value obtained from siRNAs with 2′-F at guide strand position 5 or 7 by the value obtained from siRNAs with 2′-F at guide strand position 5 or 7; if this number was <1 the negative reciprocal is shown. (D, E, F) Positive or negative values indicate increases or decreases in values when 2′-F replaces 2′-OMe at guide strand position 5 or 7.

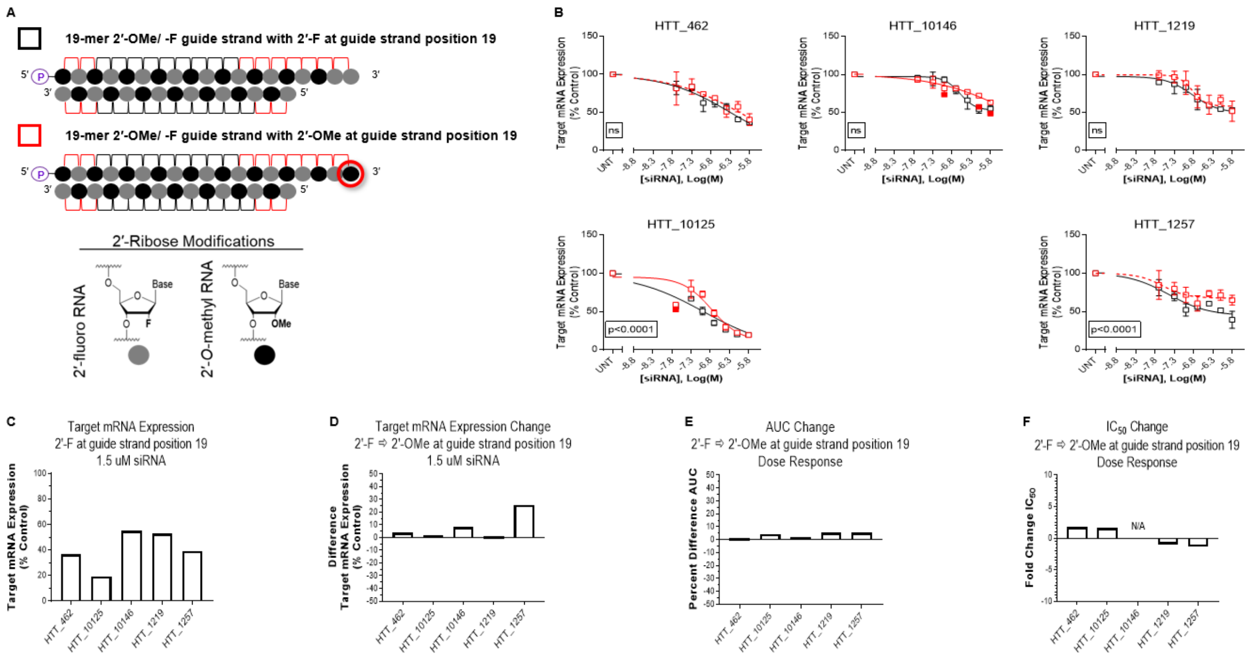

Figure S3. 2′-*O*-methyl at 3′ termini of 19-mer guide strands does not impact activity of fully modified, asymmetric siRNAs. (A) Schematic representations of chemical modification scaffolds used; symbols next to each schematic are used in graphs shown in (B). Legend shows corresponding chemical structures for 2′-ribose modifications. (B) Dose response results (n=3, mean ± SD). Target name and start site of target sequence indicated in each graph. HeLa cells were treated with siRNAs at concentrations shown for 72 hours. mRNA levels were measured using the Quantigene 2.0 RNA Assay and calculated as a percent of those from untreated cell controls. Statistical outliers were excluded from analyses, but are shown in the graphs as solid data points. Non-linear regression curves with R^2<0.8 are displayed as dashed rather than solid lines. p-values displayed on each graph calculated by two-way ANOVA. (C) Target mRNA expression with 1.5 μM of each siRNA with 2′-F at guide strand position 19. (D) Differences in target mRNA expression with 1.5 μM of each siRNA when 2′-OMe replaces 2′-F at guide strand position 19. (E) Percent differences in AUCs. (F) Fold changes in IC_{50} values, calculated by dividing the value obtained from siRNAs with 2′-OMe at guide strand position 19 by the value obtained from siRNAs with 2′-F at guide strand position 19; if this number was <1 the negative reciprocal is shown. “N/A” is shown if the Prism calculated IC_{50} value for one or both siRNAs for that sequence is greater than the top treatment dose (i.e. 1.5 μM). (D, E, F) Positive or negative values indicate increases or decreases in values when 2′-OMe replaces 2′-F at guide strand position 19.

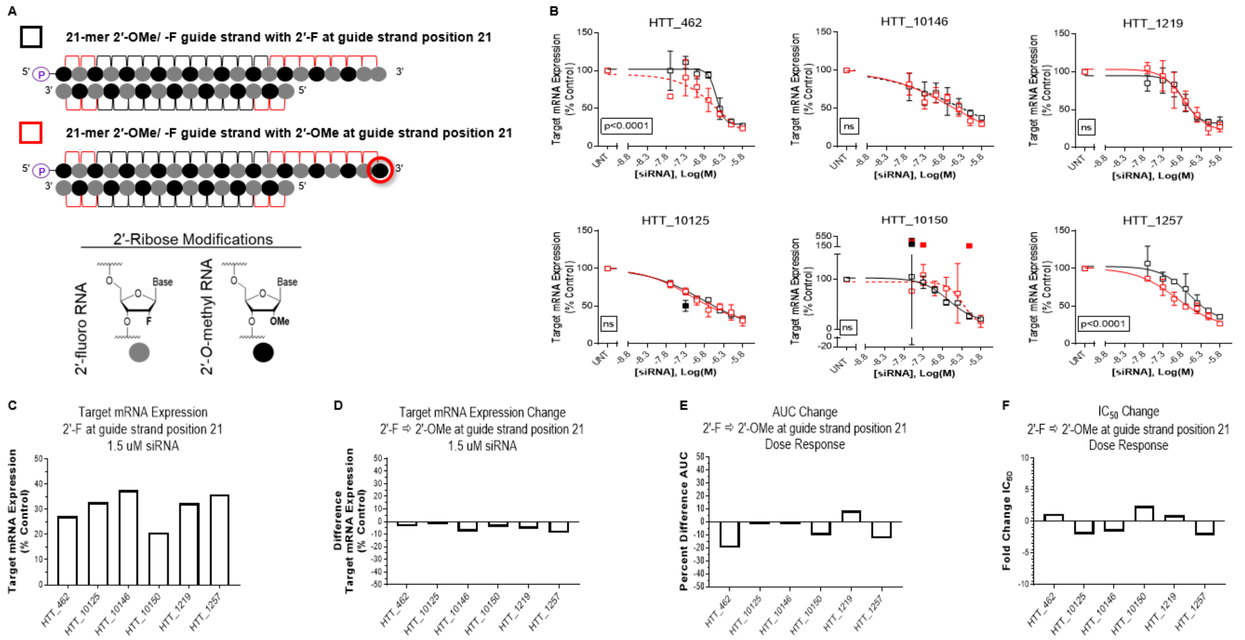

Figure S4. 2′-*O*-methyl at 3′ termini of 21-mer guide strands does not impact activity of fully modified, asymmetric siRNAs. (A) Schematic representations of chemical modification scaffolds used; symbols next to each schematic are used in graphs shown in (B). Legend shows corresponding chemical structures for 2′-ribose modifications. (B) Dose response results (n=3, mean ± SD). Target name and start site of target sequence indicated in each graph. HeLa cells were treated with siRNAs at concentrations shown for 72 hours. mRNA levels were measured using the Quantigene 2.0 RNA Assay and calculated as a percent of those from untreated cell controls. Statistical outliers were excluded from analyses, but are shown in the graphs as solid data points. Non-linear regression curves with $R^2<0.8$ are displayed as dashed rather than solid lines. p-values displayed on each graph calculated by two-way ANOVA. (C) Target mRNA expression with 1.5 μM of each siRNA with 2′-F at guide strand position 21. (D) Differences in target mRNA expression with 1.5 μM of each siRNA when 2′-OMe replaces 2′-F at guide strand position 21. (E) Percent differences in AUCs. (F) Fold changes in IC_{50} values, calculated by dividing the value obtained from siRNAs with 2′-OMe at guide strand position 21 by the value obtained from siRNAs with 2′-F at guide strand position 21; if this number was <1 the negative reciprocal is shown. (D, E, F) Positive or negative values indicate increases or decreases in values when 2′-OMe replaces 2′-F at guide strand position 21.

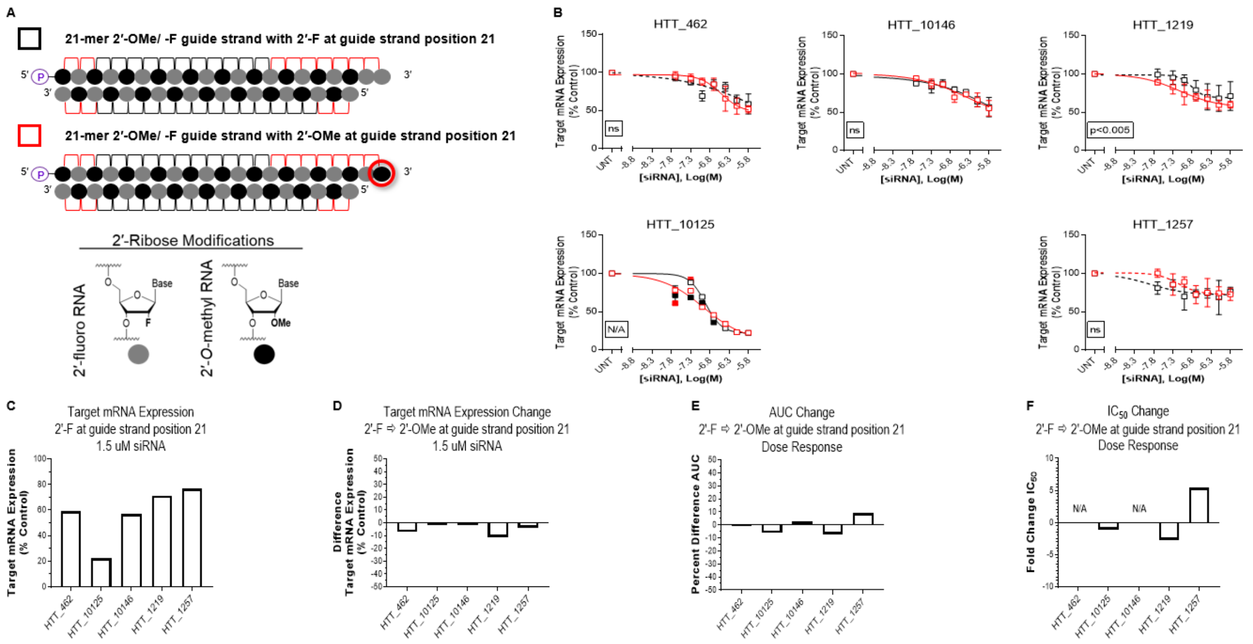

Figure S5. Sequences tested do not tolerate change from asymmetric to more symmetric 21-/19-mer siRNA structure. (A) Schematic representations of chemical modification scaffolds used; symbols next to each schematic are used in graphs shown in (B). Legend shows corresponding chemical structures for 2′-ribose modifications. (B) Dose response results (n=3, mean ± SD). Target name and start site of target sequence indicated in each graph. HeLa cells were treated with siRNAs at concentrations shown for 72 hours. mRNA levels were measured using the Quantigene 2.0 RNA Assay and calculated as a percent of those from untreated cell controls. Statistical outliers were excluded from analyses, but are shown in the graphs as solid data points. Non-linear regression curves with $R^2<0.8$ are displayed as dashed rather than solid lines. p-values displayed on each graph calculated by two-way ANOVA. Note that a p-value could not be calculated for HTT_10125 because all 3 points at the lowest test concentration for HTT_10125 siRNA with 3′-end 2′-F were excluded as statistical outliers. (C) Target mRNA expression with 1.5 µM of each siRNA with 2′-F at guide strand position 21. (D) Differences in target mRNA expression with 1.5 µM of each siRNA when 2′-OMe replaces 2′-F at guide strand position 21. (E) Percent differences in AUCs. (F) Fold changes in IC_{50} values, calculated by dividing the value obtained from siRNAs with 2′-OMe at guide strand position 21 by the value obtained from siRNAs with 2′-F at guide strand position 21; if this number was <1 the negative reciprocal is shown. “N/A” is shown if the Prism calculated IC_{50} value for one or both siRNAs for that sequence is greater than the top treatment dose (i.e. 1.5 µM). (D, E, F) Positive or negative values indicate increases or decreases in values when 2′-OMe replaces 2′-F at guide strand position 21.

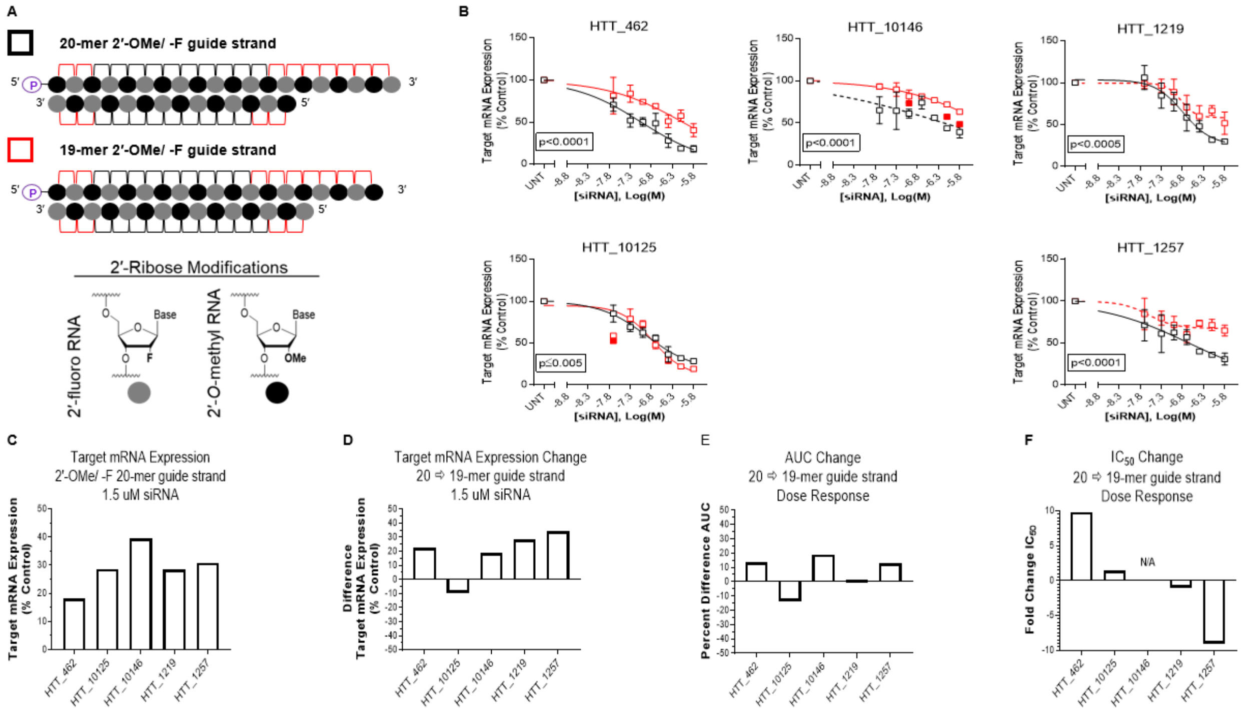

Figure S6. Sequences tested tolerate 20-/15-mer but not 19-/15-mer asymmetric siRNA structure. (A) Schematic representations of chemical modification scaffolds used; symbols next to each schematic are used in graphs shown in (B). Legend shows corresponding chemical structures for 2′-ribose modifications. (B) Dose response results (n=3, mean ± SD). Target name and start site of target sequence indicated in each graph. HeLa cells were treated with siRNAs at concentrations shown for 72 hours. mRNA levels were measured using the Quantigene 2.0 RNA Assay and calculated as a percent of those from untreated cell controls. Statistical outliers were excluded from analyses, but are shown in the graphs as solid data points. Non-linear regression curves with $R^2<0.8$ are displayed as dashed rather than solid lines. p-values displayed on each graph calculated by two-way ANOVA. (C) Target mRNA expression with 1.5 μM of each siRNA with a 20-mer guide strand and 2′-F at guide strand position 20. (D) Differences in target mRNA expression with 1.5 μM of each siRNA when 1 nucleotide is removed from the 3′ end of the guide strand. (E) Percent differences in AUCs. (F) Fold changes in IC_{50} values, calculated by dividing the value obtained from siRNAs with 19-mer guide strands by the value obtained from siRNAs with 20-mer guide strands; if this number was <1 the negative reciprocal is shown. "N/A" is shown if the Prism calculated IC_{50} value for one or both siRNAs for that sequence is greater than the top treatment dose (i.e. 1.5 μM). (D, E, F) Positive or negative values indicate increases or decreases in values when 1 nucleotide is removed from the 3′ end of the guide strand.

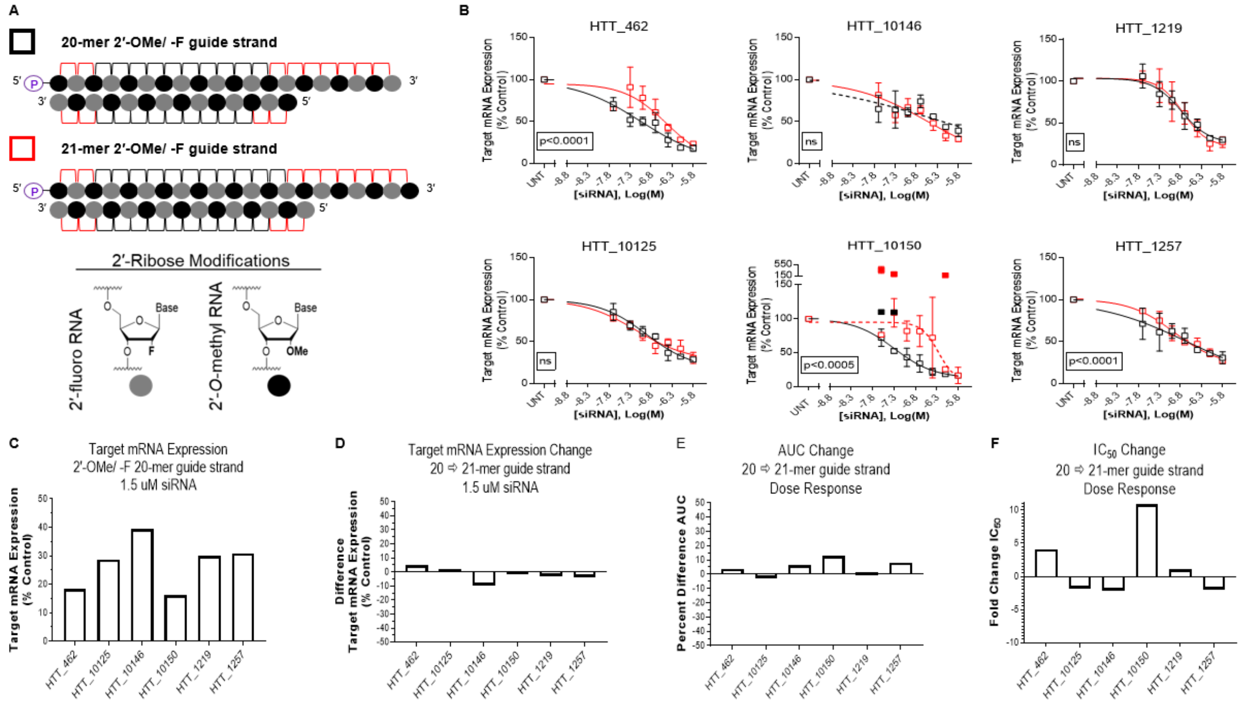

Figure S7. Sequences tested tolerate 20-/15-mer and 21-/15-mer asymmetric siRNA structure. (A) Schematic representations of chemical modification scaffolds used; symbols next to each schematic are used in graphs shown in (B). Legend shows corresponding chemical structures for 2′-ribose modifications. (B) Dose response results (n=3, mean ± SD). Target name and start site of target sequence indicated in each graph. HeLa cells were treated with siRNAs at concentrations shown for 72 hours. mRNA levels were measured using the Quantigene 2.0 RNA Assay and calculated as a percent of those from untreated cell controls. Statistical outliers were excluded from analyses, but are shown in the graphs as solid data points. Non-linear regression curves with $R^2<0.8$ are displayed as dashed rather than solid lines. p-values displayed on each graph calculated by two-way ANOVA. (C) Target mRNA expression with 1.5 µM of each siRNA with a 20-mer guide strand and 2′-F at guide strand position 20. (D) Differences in target mRNA expression with 1.5 µM of each siRNA when 1 nucleotide is added to the 3′ end of the guide strand. (E) Percent differences in AUCs. (F) Fold changes in IC_{50} values, calculated by dividing the value obtained from siRNAs with 21-mer guide strands by the value obtained from siRNAs with 20-mer guide strands; if this number was <1 the negative reciprocal is shown. (D, E, F) Positive or negative values indicate increases or decreases in values when 1 nucleotide is added to the 3′ end of the guide strand.

Supplemental Tables

Table S1. Values from Figure S1. When looking at differences, positive values indicate an increase in values with the application of the indicated modification change. IC_{50} fold change was calculated by dividing the value obtained with the indicated modification change by the value obtained without it. However, if this number was <1 the negative reciprocal is listed (e.g. 0.75, or a drop of 25% from the original value is reported as -1.3 fold change).

**Guide Strand Modification Pattern**	**2′-OMe/ -F**	**2′-OMe at Position 20**	**+ 2′-OMe at Position 20**
**Target**	**% Target mRNA Expression with 1.5 uM siRNA**		**Difference % Target mRNA Expression with 1.5 uM siRNA**
sFLT1-i13_2283	18.9	32.3	13.4
**Target**	**Area Under Curve**		**% Difference Area Under Curve**
sFLT1-i13_2283	622.4	760.0	19.9
**Target**	**IC_{50} Value (nM)**		**Fold Change IC_{50} Value**
sFLT1-i13_2283	204.1	292.8	1.4

Table S2. Values from Figure S2. When looking at differences, positive or negative values indicate an increase or decrease in values with the application of the indicated modification change. IC_{50} fold change was calculated by dividing the value obtained with the indicated modification change by the value obtained without it. However, if this number was <1 the negative reciprocal is listed (e.g. 0.75, or a drop of 25% from the original value is reported as -1.3 fold change).

**Guide Strand Modification Pattern**	**2′-OMe at Position 20**	**2′-OMe at Position 20 and 2′-F at Position 5**	**2′-OMe at Position 20 and 2′-F at Position 7**	**+ 2′-F at Position 5**	**+ 2′-F at Position 7**
**Target**	**% Target mRNA Expression with 1.5 uM siRNA**			**Difference % Target mRNA Expression with 1.5 uM siRNA**	
sFLT1-i13_2283	32.3	7.3	19.7	-25.0	-12.7
sFLT1-e15a_2519	23.0	19.0	46.3	-4.0	23.3
**Target**	**Area Under Curve**			**% Difference Area Under Curve**	
sFLT1-i13_2283	605.4	399.6	623.5	-41.0	2.9
sFLT1-e15a_2519	700.5	493.6	635.5	-34.7	-9.7
**Target**	**IC_{50} Value (nM)**			**Fold Change IC_{50} Value**	
sFLT1-i13_2283	78.7	12.0	149.7	-6.6	1.9
sFLT1-e15a_2519	110.1 [a]	55.5	135.5	-2.0	1.2

[a]The R^2 value for the fitted curve used to calculate the IC_{50} value <0.8.

Table S3. Values from Figure S3. When looking at differences, positive or negative values indicate an increase or decrease in values with the application of the indicated modification change. IC_{50} fold change was calculated by dividing the value obtained with the indicated modification change by the value obtained without it. However, if this number was <1 the negative reciprocal is listed (e.g. 0.75, or a drop of 25% from the original value is reported as -1.3 fold change). "N/A" = not applicable.

**Guide Strand Modification Pattern**	**2′-F at Position 19**	**2′-OMe at Position 19**	**+ 2′-OMe at Position 19**
**Target**	**% Target mRNA Expression with 1.5 µM siRNA**		**Difference % Target mRNA Expression with 1.5 uM siRNA**
HTT_462	36.6	40.6	4.0
HTT_10125	19.4	19.3	-0.2
HTT_10146	55.1	58.5	3.4
HTT_1219	52.7	51.8	-0.9
HTT_1257	39.2	65.0	25.8
**Target**	**Area Under Curve**		**% Difference Area Under Curve**
HTT_462	600.6	608.3	1.3
HTT_10125	493.5	515.6	4.4
HTT_10146	666.3	665.8	-0.1
HTT_1219	638.1	674.4	5.5
HTT_1257	592.9	627.2	5.6
**Target**	**IC_{50} Value (nM)**		**Fold Change IC_{50} Value**
HTT_462	506.7	893.8[a]	1.8
HTT_10125	106.9	171.3	1.6
HTT_10146	252.8	>1500[b]	N/A
HTT_1219	146.8	146.7[a]	-1.0
HTT_1257	35.61	25.01[a]	-1.4

[a]The R^2 value for the fitted curve used to calculate the IC_{50} value <0.8.
[b]The Prism calculated IC_{50} value is greater than the top treatment dose (i.e. 1.5 µM) and is therefore excluded from analysis (see Materials & Methods).

Table S4. Dose response data at top three treatment concentrations for HTT_10125 with 20- and 19-mer guide strands. When looking at differences, positive or negative values indicate an increase or decrease in values with the application of the indicated modification change.

	**Guide Strand Modification Pattern**	**2′-F at 3′ end of Guide Strand**	**2′-OMe at 3′ end of Guide Strand**	**+ 2′-OMe at 3′ end of Guide Strand**	
**siRNA**	**Dose (μM)**	**% Target mRNA Expression**		**Difference % Target mRNA Expression**	**Average Difference % Target mRNA Expression**
HTT_10125 with 20-mer Guide Strand	1.5	28.7	33.4	4.7	7.5
	0.75	32.2	39.5	7.3	
	0.375	36.7	47.3	10.6	
HTT_10125 with 19-mer Guide Strand	1.5	19.4	19.3	-0.2	1.8
	0.75	20.0	22.3	2.3	
	0.375	26.3	29.7	3.4	

Table S5. Values from Figure S4. When looking at differences, positive or negative values indicate an increase or decrease in values with the application of the indicated modification change. IC_{50} fold change was calculated by dividing the value obtained with the indicated modification change by the value obtained without it. However, if this number was <1 the negative reciprocal is listed (e.g. 0.75, or a drop of 25% from the original value is reported as -1.3 fold change).

**Guide Strand Modification Pattern**	**2′-F at Position 21**	**2′-OMe at Position 21**	**+ 2′-OMe at Position 21**
**Target**	**% Target mRNA Expression with 1.5 uM siRNA**		**Difference % Target mRNA Expression with 1.5 uM siRNA**
HTT_462	27.4	23.4	-4.0
HTT_10125	32.8	30.7	-2.1
HTT_10146	37.7	29.7	-8.0
HTT_10150	20.9	16.4	-4.5
HTT_1219	32.6	27.0	-5.6
HTT_1257	36.0	27.0	-9.0
**Target**	**Area Under Curve**		**% Difference Area Under Curve**
HTT_462	674.4	551.6	-20.0
HTT_10125	586.0	576.9	-1.6
HTT_10146	585.6	587.3	0.3
HTT_10150	665.4	601.6	-10.1
HTT_1219	608.1	663.3	8.7
HTT_1257	681.4	599.8	-12.7
**Target**	**IC_{50} Value (nM)**		**Fold Change IC_{50} Value**
HTT_462	321.8	372.9[a]	1.2
HTT_10125	149.1	70.2	-2.1
HTT_10146	489.5[a]	310.5	-1.6
HTT_10150	228.5	546.1[a]	2.4
HTT_1219	178.8	181.1	1.0
HTT_1257	248.6	113.6	-2.2

[a]The R^2 value for the fitted curve used to calculate the IC_{50} value <0.8.

Table S6. Values from Figure S5. When looking at differences, positive or negative values indicate an increase or decrease in values with the application of the indicated modification change. IC_{50} fold change was calculated by dividing the value obtained with the indicated modification change by the value obtained without it. However, if this number was <1 the negative reciprocal is listed (e.g. 0.75, or a drop of 25% from the original value is reported as -1.3 fold change). "N/A" = not applicable.

**Guide Strand Modification Pattern**	**2′-F at Position 21**	**2′-OMe at Position 21**	**+ 2′-OMe at Position 21**
**Target**	**% Target mRNA Expression with 1.5 uM siRNA**		**Difference % Target mRNA Expression with 1.5 uM siRNA**
HTT_462	59.0	51.9	-7.1
HTT_10125	22.3	22.6	0.3
HTT_10146	56.8	55.2	-1.6
HTT_1219	71.2	59.8	-11.4
HTT_1257	76.5	72.9	-3.6
**Target**	**Area Under Curve**		**% Difference Area Under Curve**
HTT_462	660.7	666.6	0.9
HTT_10125	601.9	565.8	-6.2
HTT_10146	644.6	661.9	2.6
HTT_1219	684.4	637.7	-7.1
HTT_1257	622.7	684.4	9.4
**Target**	**IC_{50} Value (nM)**		**Fold Change IC_{50} Value**
HTT_462	>1500[a, b]	309.3	N/A
HTT_10125	121.9	109.1	-1.1
HTT_10146	>1500[b]	424.2	N/A
HTT_1219	151.3[a]	56.2	-2.7
HTT_1257	12.87[a]	69.6[a]	5.4

[a]The R^2 value for the fitted curve used to calculate the IC_{50} value <0.8.
[b]The Prism calculated IC_{50} value is greater than the top treatment dose (i.e. 1.5 μM) and is therefore excluded from analysis (see Materials & Methods).

Table S7. Values from Figure S6. When looking at differences, positive or negative values indicate an increase or decrease in values with the application of the indicated modification change. IC_{50} fold change was calculated by dividing the value obtained with the indicated modification change by the value obtained without it. However, if this number was <1 the negative reciprocal is listed (e.g. 0.75, or a drop of 25% from the original value is reported as -1.3 fold change). "N/A" = not applicable.

**Guide Strand Modification Pattern**	**2′-OMe/ -F 20-mer**	**2′-OMe/ -F 19-mer**	**- 1 Nucleotide**
**Target**	**% Target mRNA Expression with 1.5 uM siRNA**		**Difference % Target mRNA Expression with 1.5 uM siRNA**
HTT_462	18.3	40.6	22.3
HTT_10125	28.7	19.3	-9.4
HTT_10146	39.5	58.5	19.0
HTT_1219	30.1	58.5	28.4
HTT_1257	30.9	65.0	34.1
**Target**	**Area Under Curve**		**% Difference Area Under Curve**
HTT_462	531.1	608.3	13.6
HTT_10125	592.9	515.6	-13.9
HTT_10146	550.2	665.8	19.0
HTT_1219	666.7	674.4	1.1
HTT_1257	553.0	627.2	12.6
**Target**	**IC_{50} Value (nM)**		**Fold Change IC_{50} Value**
HTT_462	91.6	893.8	9.8
HTT_10125	122.9	171.3	1.4
HTT_10146	643.3[a]	>1500[b]	N/A
HTT_1219	164.0	146.7[a]	-1.1
HTT_1257	227.3	25.0[a]	-9.1

[a]The R^2 value for the fitted curve used to calculate the IC_{50} value <0.8.
[b]The Prism calculated IC_{50} value is greater than the top treatment dose (i.e. 1.5 μM) and is therefore excluded from analysis (see Materials & Methods).

Table S8. Values from Figure S7. When looking at differences, positive or negative values indicate an increase or decrease in values with the application of the indicated modification change. IC_{50} fold change was calculated by dividing the value obtained with the indicated modification change by the value obtained without it. However, if this number was <1 the negative reciprocal is listed (e.g. 0.75, or a drop of 25% from the original value is reported as -1.3 fold change).

**Guide Strand Modification Pattern**	**2′-OMe/ -F 20-mer**	**2′-OMe/ -F 21-mer**	**+ 1 Nucleotide**
**Target**	**% Target mRNA Expression with 1.5 uM siRNA**		**Difference % Target mRNA Expression with 1.5 uM siRNA**
HTT_462	18.3	23.4	5.1
HTT_10125	28.7	30.7	2.0
HTT_10146	39.5	29.7	-9.7
HTT_10150	16.2	16.4	0.1
HTT_1219	30.1	27.0	-3.1
HTT_1257	30.9	27.0	-3.9
**Target**	**Area Under Curve**		**% Difference Area Under Curve**
HTT_462	531.1	551.6	3.8
HTT_10125	592.9	576.9	-2.7
HTT_10146	550.2	587.3	6.5
HTT_10150	528.8	601.6	12.9
HTT_1219	666.7	663.3	-0.5
HTT_1257	553.0	599.8	8.1
**Target**	**IC_{50} Value (nM)**		**Fold Change IC_{50} Value**
HTT_462	91.61	372.9	4.1
HTT_10125	122.9	70.2	-1.8
HTT_10146	643.3[a]	310.5	-2.1
HTT_10150	50.3	546.1[a]	10.9
HTT_1219	164.0	181.0	1.1
HTT_1257	227.3	113.6	-2.0

[a]The R^2 value for the fitted curve used to calculate the IC_{50} value <0.8.

Table S9. Oligonucleotides examined in PyMol. ‘P’ denotes phosphate; ‘VP-T’ denotes 2′-O-MOE-thymidine-(E)-5′-vinylphosphonate; ‘#’ denotes phosphorothioate instead of phosphodiester; ‘m’ denotes 2′-O-methyl; ‘f’ denotes 2′-fluoro; ‘me’ denotes 2′-methoxyethyl; ‘A’ denotes Adenine; ‘U’ denotes Uracil; ‘G’ denotes Guanosine; ‘C’ denotes Cysteine.

**PDB Reference Number**	**Guide Strand Sequence with Chemical Modification Pattern**
4W5N, 4W5Q	P(U)(U)(C)(A)(C)(A)(U)(U)(G)(C)(C)(C)(A)(A)(G)(U)(C)(U)(C)(U)(U)
5JS2	VP-T#(fU)#(mA)(fU)#(mC)(fU)#(mA)(fU)#(mA)(fA)#(mU)(fG)#(mA)(fU)#(mC)#(fA)#(mG)#(fG)#(mU)#(meA)#(meA)
3MJ0	(C)(G)(U)(U)(A)(C)(G)(C)(mU)

Table S10. Oligonucleotide duplexes used in in vitro efficacy and thermal melt studies. Information for each duplex is boxed with a thick outside border. ‘P’ denotes phosphate; ‘#’ denotes phosphorothioate instead of phosphodiester; ‘m’ denotes 2′-O-methyl; ‘f’ denotes 2′-fluoro; 'r' denotes 2′-OH; ‘A’ denotes Adenine; ‘U’ denotes Uracil; ‘G’ denotes Guanosine; ‘C’ denotes Cysteine; ‘TegChol’ denotes Triethylene Glycol linker + Cholesterol conjugate.
